# Person-centred care models for institutionalised older adults requiring palliative care: an integrative review

**DOI:** 10.1186/s12877-026-07795-y

**Published:** 2026-07-07

**Authors:** Lucinda Marques, Bruno Morgado, Alícia Oliveira, Ana Lazana, Lara Guedes Pinho, Isabel Bico, Manuel José Lopes

**Affiliations:** 1 Nursing Department, Universidade de Évora, Évora, 7000-811 Portugal; 2 Comprehensive Health Research Centre (CHRC), Évora, 7000-811 Portugal; 3 Department of Nursing, Universitat Rovira E Virgilli, Tarragona, Spain; 4 Polytechnic Institute of Portalegre, Portalegre, Portugal; 5 Unidade Local de Saúde Alentejo Central, Évora, 7000 - 811 Portugal; 6 Escola Superior de Saúde, Instituto Politécnico de Viana Do Castelo, Viana Do Castelo, 4900-314 Portugal; 7 LA REAL - Associated Laboratory in Translation and Innovation Towards Global Health, University of Évora, Évora, Portugal

**Keywords:** Person-centred care, Palliative care, Institutionalised older adults, Interdisciplinary collaboration, Communication, Long-term care, Human dignity, Healthcare ethics, Training, Transdisciplinary

## Abstract

**Background:**

This review explores strategies for implementing person-centred care (PCC) for older adults in institutional care settings with palliative care needs. Such settings present complex challenges, including loss of autonomy, multiple chronic conditions and psychological or existential distress. Integrating PCC principles with palliative care is an appropriate approach as it promotes shared decision-making, dignity, comfort and well-being. This approach contributes to individual satisfaction and the sustainability of care systems.

**Aim:**

To synthesise the scientific evidence on person-centred care models for institutionalised older adults in need of palliative care.

**Materials and methods:**

A literature review was conducted using five databases. The initial search covered the period from December 2017 to August 2022. An additional search was performed between September 2022 and June 2025 to ensure coverage. The search strategy incorporated Medical Subject Headings (MeSH) terms and equivalent descriptors, including palliative care, end-of-life care, palliative nursing, long-term care, nursing homes, residential care, person-centred care, patient-centred care and client-centred care. The review was conducted in accordance with the PRISMA methodology and was registered in the PROSPERO database (CRD42022373388).

**Results:**

A total of 201 records were identified, of which nine studies met the inclusion criteria and were included in the final synthesis. Effective implementation of PCC requires more than technical proficiency; it demands advanced communication, interpersonal, reflective and analytical skills, which are rooted in ethical reasoning and collaborative practice. Major barriers included insufficient staff competencies, limited training, resource constraints, and institutional rigidity. Facilitators included motivational leadership, continuous professional development, interdisciplinary collaboration, and organisational support. These findings are consistent with the Person-Centred Practice Framework developed by McCormack and McCance and the work of the Gothenburg Centre for Person-Centred Care. Both frameworks emphasise co-constructed care planning and respect for patient narratives as core principles of PCC.

**Conclusion:**

This integrative review provides a comprehensive synthesis of the conditions that enable PCC implementation in long-term care. The findings emphasise the importance of ethical care practices, structural support, and ongoing investment in workforce development. The study reinforces the relevance of transdisciplinary, human rights–based models and offers practical recommendations for care delivery, education, and policy design.

**Supplementary Information:**

The online version contains supplementary material available at https://doi.org/10.1186/s12877-026-07795-y.

## Introduction

Technical, scientific, and socio-economic advances over recent decades have led to significant improvements in public health, reflected in increased life expectancy. However, declining birth and mortality rates have been major contributors to population ageing, one of the most significant social changes of the twenty-first century, with far-reaching implications across all sectors of society [1–3].

The global population aged 60 and over is projected to double from 962 million in 2017 to 2.1 billion by 2050, while the population aged 80 and over is expected to triple [3]. According to the Organisation for Economic Co-operation and Development (OECD), the proportion of individuals aged 65 and over will rise from 18% in 2021 to 27% by 2050. In countries such as Portugal, this proportion will exceed one-third, with more than one in eight people being 80 or older [4]. Despite gains in longevity, this trend has not been accompanied by equivalent increases in healthy life expectancy or significant health improvements [5].

The coexistence of chronic illness, functional decline, and caregiver shortages contributes significantly to the institutionalisation of older adults [6], raising major public health concerns due to their complex, multidimensional needs [5, 7–10]. Many long-term care residents now present with advanced frailty, multiple chronic conditions, and polypharmacy, complicating clinical management [11–15].

Erving Goffman's concept of the "total institution" (1961) remains central to understanding the impact of institutionalisation on autonomy and personal identity [16]. Despite policy and technological progress, institutional care remains heterogeneous and fragmented, shaped by each country's healthcare system, social care resources, and funding models [17]. In this context, redefining "health needs" becomes essential, especially given their multidimensional nature, encompassing physical, psychological, social, therapeutic, and existential aspects [15, 18–20]. Ageing societies demand integrated, sustainable responses. These require complex thinking and transdisciplinary strategies, moving beyond siloed approaches and fostering co-created, evidence-informed solutions [5, 21–23].

A holistic paradigm is essential, one that considers not only health and disease but also personal values, beliefs, preferences, and life goals [24, 25]. Person-centred care (PCC) and palliative care have emerged as complementary models that respond to this challenge, aligning with frameworks such as McCormack and McCance’s person-centred practice theory and the University of Gothenburg’s Centre for Person-Centred Care (GPCC) [19, 20, 24, 26–28].

Drawing on Carl Rogers’ humanistic psychology, PCC emphasises empathy, acceptance, and autonomy throughout the life course [29], while Thomas's transformative model of ageing introduced a more dynamic and active vision of later life [30]. Studies increasingly support the benefits of PCC, especially in populations with multimorbidity and at the end of life by incorporating individual values and preferences into care planning, improving both outcomes and patient experience [19, 20, 31, 32]. Supporting this paradigm shift requires healthcare professionals trained not only in technical but also in relational and ethical competencies [19, 20]. In this context, palliative care plays a critical role, ensuring an integrated and compassionate approach to suffering in all its dimensions, including physical, emotional, social, and spiritual [12, 33–38]. According to the World Health Organization (WHO, 2023), palliative care improves quality of life for patients and families through early identification and appropriate management of distressing symptoms, while affirming life and viewing death as a natural process [27, 39].

Integrating palliative care into long-term care institutions is increasingly recognised as best practice for managing frailty and chronic disease in ageing populations [11, 27, 28, 38]. Beyond addressing symptoms, it fosters dignity, respects patient preferences, and engages family members. However, implementing these changes requires skilled teams and organizational models that support interdisciplinary collaboration [20, 28, 40–43].

Current evidence indicates that a significant proportion of institutional care is provided by personnel who lack the necessary qualifications, frequently without higher education or specialized training. This situation compromises the quality of care and places strain on health systems [44–47]. According to OECD data, less than 25% of long-term care staff possess higher education qualifications, with over 70% working as uncertified caregivers [46]. This lack of training contributes to suboptimal practices and increased use of emergency services by institutionalized residents [28, 40, 48, 49].

Structured education in palliative care and PCC is thus essential not only to meet the demands of multimorbidity and ageing but also to promote compassionate, efficient care. International bodies such as the European Association for Palliative Care (EAPC) and WHO have called for the integration of these fields into both undergraduate curricula and continuing professional education [5]. Theoretical frameworks like McCormack and McCance’s Middle Range Theory and the GPCC model provide strong foundations for implementing PCC through active listening, collaborative care planning, and a focus on dignity and well-being [24, 26].

Moreover, discussions on sustainability, equity, and healthcare efficiency underscore the importance of these approaches. Research confirms that palliative and person-centred care reduce avoidable hospitalisations, improve patient and caregiver satisfaction, and support more sustainable healthcare systems [15, 35, 50–52]. Investing in training and implementing these care models is not only an ethical obligation, but also a strategic necessity when addressing today's demographic and clinical challenges.

In view of the increasing number of institutionalised older adults with complex and multidimensional care needs, particularly those related to chronic illness and end-of-life trajectories, there is an urgent need to rethink and strengthen care models within long-term care settings, especially in the context under study. However, current practices remain fragmented, and the evidence on person-centred palliative approaches is limited and heterogeneous. The aim of this review is to synthesise the scientific evidence on care strategies for institutionalised older adults with palliative care needs.

## Materials and methods

This study was developed as an integrative review of the literature, with the aim of synthesising empirical evidence on models of person-centred care (PCC) for institutionalised older adults with palliative needs.

### Search strategy

Relevant publications were identified through comprehensive searches of the following electronic databases: CINAHL Plus with Full Text, MedicLatina, MEDLINE with Full Text, Academic Search Complete, and Psychology and Behavioural Sciences Collection.

The search strategy combined Medical Subject Headings (MeSH) and equivalent free-text terms related to both *palliative care* and *person-centred approaches*. Key terms included: *palliative care*, *end-of-life care*, *palliative nursing*, *long-term care*, *nursing homes*, *residential care*, *person-centred care*, *patient-centred care*, and *client-centred care*.

The initial search covered studies published between December 2017 and August 2022, a period selected to capture recent developments following global health policy reforms promoting integrated and humanised long-term care. To ensure the review remained current and aligned with PRISMA 2020 recommendations, an updated search was conducted between September 2022 and June 2025 using the same search strategy and eligibility criteria as the initial search.

**Fig. 1:**
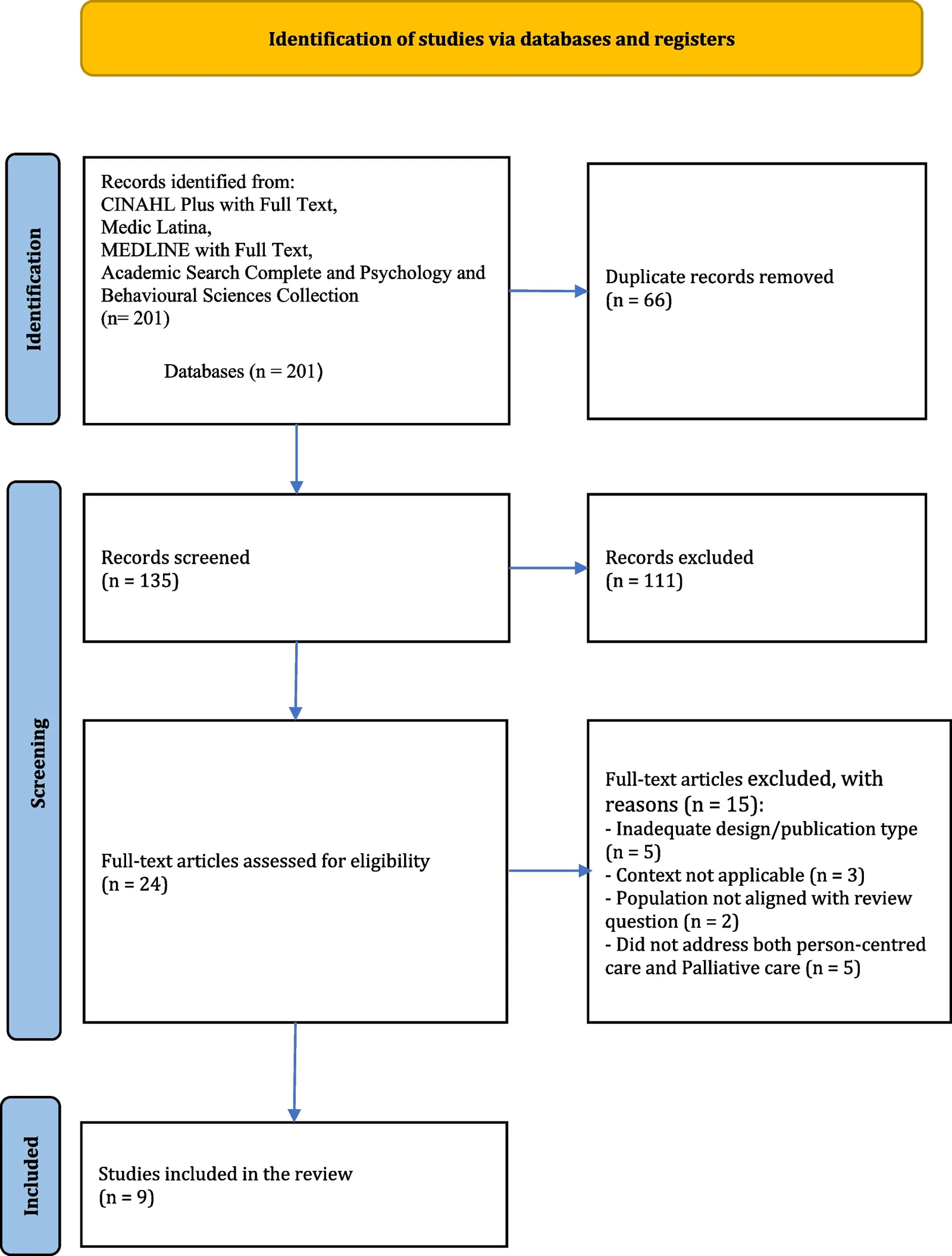
PRISMA 2020 flow diagram of the study selection process

All records identified across the different search phases were exported and combined, and duplicates were removed prior to screening. The study selection process followed PRISMA 2020 guidelines and is presented in Fig. 1.

This review was conducted in accordance with the Preferred Reporting Items for Systematic Reviews and Meta-Analyses (PRISMA 2020) guidelines and was registered in the International Prospective Register of Systematic Reviews (PROSPERO) (Registration No. CRD42022373388) [33].

### Eligibility criteria

The PICo framework was applied to guide the inclusion and exclusion process (P = Population, I = Interest, Co = Context) [43], ensuring a methodical, transparent, and replicable selection process. The inclusion and exclusion criteria, detailed according to the PICo structure, are summarised in Table 1.

**Table 1:** Inclusion criteria according to the PICo framework

Element	Description
P (Population)	Institutionalised older adults (≥ 60 years) living in nursing homes
I (Interest)	Palliative care actions or person-centred care interventions addressing palliative needs
Co (Context)	Nursing homes or long-term care institutions (public or private, long- or short-term)

The overarching research question guiding this study was:

Which care models or interventions focused on institutionalised older adults recognise, consider, and respond to their palliative care needs?

#### Inclusion criteria

Studies were included if they:

- Involved institutionalised older adults aged 60 years or over, living in public or private nursing homes (long-or short-term).
- Described palliative care interventions or approaches consistent with a palliative care philosophy (diagnostic assessment, needs identification, care planning, or symptom relief).
- Adopted a person-centred care perspective, addressing autonomy, shared decision-making, and holistic well-being.

#### Exclusion criteria

Studies were excluded if they:

- Were conducted in hospital or primary care settings.
- Focused on non-institutionalised populations.
- Were systematic reviews, meta-analyses, or theoretical reflections lacking empirical data.

### Study selection and data collection

A total of 201 records were initially identified and imported into Mendeley Reference Manager. Following the removal of 66 duplicates, 135 records were screened by two independent reviewers based on title and abstract, applying the predefined eligibility criteria. Disagreements were resolved by a third reviewer.

Of these, 111 records were excluded for not meeting the inclusion criteria. Twenty-four reports were assessed in full-text for eligibility. Fifteen reports were excluded after full-text review for the following reasons: inadequate study design or publication type (*n* = 5), non-applicable context (*n* = 3), population not aligned with the review question (*n* = 2), or lack of focus on both person-centred care and palliative care (*n* = 5).

A total of nine studies met all inclusion criteria and were included in the final synthesis. The study selection process is presented in Fig. 1, in accordance with the PRISMA 2020 flow diagram.

### Quality appraisal and data analysis

The methodological quality of each study was assessed using the Joanna Briggs Institute (JBI) Critical Appraisal Checklist [53], which evaluates potential sources of bias in study design, conduct, and analysis. The results of the quality appraisal are presented in Supplementary File 1.

Thematic synthesis was conducted using an inductive approach. Following data extraction, findings from each study were systematically coded to identify recurrent concepts related to person-centred care in palliative contexts. Codes were iteratively compared and grouped into higher-order categories, which were subsequently refined into overarching themes and competencies. This process involved constant comparison across studies to enhance analytical rigour and ensure consistency. The final themes represent shared patterns across the included studies and are summarised in Table 2 and further described in the Results section.

**Table 2:** Analysis of the selected studies

Publication and year	Country/Setting	Aim/Focus	Participants/Design	Data collection method	Main Findings (Paraphrased Summary)	Themes/competencies relevant to person-centred care of institutionalised older adults with palliative needs
Gonella et al. (2021) [18]	Italy/13 nursing homes	To examine nurses’ experiences and perceptions regarding communication about end-of-life issues with residents and families	14 nurses/Qualitative descriptive study	Semi-structured interviews	A few factors affecting end-of-life communication were identified, including the culture of care, staff–resident–family relationships, and the shift from curative to palliative goals. Effective communication is key to improving shared understanding and ensuring that care plans are informed by the preferences of patients and their families	• Communication skills • Empathy • Shared decision-making • Recognition of preferences
Holmberg & Godskesen (2022) [54]	Sweden/Urban nursing home	To explore ethical dimensions and moral sensitivity in encounters between residents and nursing staff during physical care	12 residents, 16 nursing assistants/Focused ethnography	Participant observation and interviews	Highlighted moral sensitivity and genuine engagement as essential to dignified care. Barriers included lack of resources and communication; facilitators were empathy, dialogue, and ethical reflection	• Ethical Awareness • Moral Sensitivity • Communication • Reflective Practice
Saarnio et al. (2019) [55]	Sweden/Two nursing homes	To identify strategies used by staff to promote a sense of “at-homeness” in residents receiving palliative care	13 residents, 14 care staff/Interpretive descriptive study	Observations and interviews	The practices identified as key factors as fostering a sense of "at-homeness" include establishing a personal connection with residents, maintaining integrity, fostering family-like relationships, ensuring continuity and encouraging reflective teamwork	• Respect For Integrity • Reliability • Teamwork • Comfort Promotion
Saarnio et al. (2017) [56]	Sweden/Non-profit nursing home	To explore how daily routines and relationships create a sense of “at-homeness” for residents in long-term care	10 healthcare professionals/Interpretive description	Focus group discussions	Emphasised the importance of continuity, personal knowledge of residents, and relational care practices to maintain dignity and identity	• Continuity • Personalisation • Relational Care • Resident Identity
Bökberg et al. (2019) [57]	Sweden/Nine nursing homes	To evaluate the effects of staff education on perceptions of person-centred palliative care	365 healthcare staff/Quasi-experimental design	Structured questionnaires (pre and post-test)	At the initial stage of the study, both groups demonstrated high levels of performance. However, after the intervention, there was only a mild improvement that had been measured. Management and organisational support have been identified as the main factors in sustaining PCC	• Leadership Engagement • Organisational Support • Team Culture
Schenell et al. (2020) [58]	Sweden/Forty residential care facilities	To co-develop a model that promotes self-determination and autonomy in residential care	Mixed group (staff, residents, managers)/Participatory design	Focus groups and workshops	The "In my way, at my pace, with the help of you" model was produced, with a view to reinforcing relational autonomy through trust, respect and shared decision-making	• Autonomy • Trust • Empowerment • Collaborative Care
Kastbom et al. (2019) [59]	Sweden/Nine nursing homes	To explore healthcare professionals’ experiences with advance care planning (ACP)	25 professionals (physicians, nurses)/Qualitative study	Individual interviews	Four phases of ACP identified: exploring preferences, integrating views, documenting, and revisiting plans. Highlighted the need for collaborative ACP and clear communication	• Advance Care Planning; Communication • Ethical Discussion
Doody et al. (2024) [60]	Ireland/Twenty-five nursing homes	To examine perceptions of residents, relatives, and staff on palliative and person-centred care provision	118 participants (residents, family members, staff)/Quantitative cross-sectional study	Structured questionnaires	Reported overall positive experiences but noted communication gaps and resource limitations. Underlined the ethical duty to uphold residents’ and professionals’ human rights	• Communication • Human Rights • Ethical Leadership • Staff Education
Mikaelsson et al. (2025) [61]	Sweden/Five residential care homes	To explore residents’ and families’ experiences of proactive end-of-life conversations	11 residents, 8 relatives/Qualitative interview study	Semi-structured interviews	Identified benefits such as open dialogue, shared understanding, emotional security, and relationship strengthening. Promoted early discussions as a core element of PCC	• Open Communication • Trust • Family Involvement • Emotional Preparedness

Summary of the included studies, highlighting methodological characteristics, key findings, and competencies relevant to person-centred care for institutionalised older adults with palliative care needs

## Results

The results are presented in two sections: the first provides an overview of the studies included in this integrative review, and the second outlines the key aspects of person-centred care (PCC) as they relate to institutionalised older adults with palliative care needs.

### Overview of included studies

A total of nine studies were included in the review [18, 54–61], all of which were conducted in nursing home settings. Despite variations in methodological approach and context, the studies shared a common aim: to improve or evaluate care models centred on the needs of institutionalised older adults requiring palliative care [18, 54, 57–60]. Seven of the studies were carried out in Sweden [54–59, 61], one in Italy [18], and one in Ireland [60]. The settings included both public and private institutions, urban and rural environments, and varied between short- and long-term residential care [54–56, 60].

Methodologically, two studies followed a quantitative approach, one of which included a control group [57, 60], while seven employed qualitative designs [18, 54–56, 58, 59, 61]. Among the qualitative studies, one adopted an ethnographic approach [54], while the remaining six used descriptive or interpretive qualitative methodologies [18, 55, 56, 58, 59, 61].

Four studies explored the perspectives of older residents [54, 55, 60, 61], two of which also included the views of family members [60, 61], the remaining studies focused primarily on healthcare professionals' experiences [18, 56, 57, 59].

The methodological quality of the included studies was assessed using the Joanna Briggs Institute checklist [53], which evaluates potential bias in study design, conduct, and analysis. Despite methodological heterogeneity, the studies consistently reflected key principles of person-centred and palliative care, including respect for individual values and preferences [18, 55, 56], promotion of autonomy [58, 59], involvement of families and professionals in shared decision-making [18, 59–61], and a holistic approach to care [54, 55, 60]. A detailed summary of the included studies, outlining their aims, designs, methods, and key findings, is presented in Table 2.

### Key aspects of person-centred care

Across the studies, several core principles of person-centred care (PCC) were consistently identified. These included respect for individual values and care goals [18, 55, 56], promotion of autonomy and self-determination [58, 59], involvement of older adults, families, and caregivers in shared decision-making [18, 59–61], comprehensive and integrated approaches to care [54, 55, 60], relationship-based and humanised care practices [54–56], a consistent focus on quality of life [55, 60], and the importance of interdisciplinary teamwork to ensure coordinated care [57, 59].

Seven of the nine studies highlighted the importance of communication and relational skills for identifying residents’ care needs and engaging stakeholders in care planning [18, 54, 55, 59–61]. Notably, Mikaelsson et al. [61] explored the experiences of residents and families regarding proactive end-of-life conversations, demonstrating that early, intentional discussions foster trust, reduce anxiety, and reinforce ethical decision-making within residential settings [61].

Two studies emphasised ethical principles and human rights as foundational dimensions of care [54, 60]. However, only three studies recommended structured staff training [54, 57, 60], and just one explicitly suggested including managers in such initiatives to strengthen leadership in palliative care implementation [57].

Barriers to implementing person-centred palliative care included lack of formal staff training and qualifications [57, 60], limited leadership involvement [57], resource constraints and inadequate institutional support [54, 60], ethical challenges and communication breakdowns, particularly in complex care situations [54, 59].

Facilitators identified across the included studies included motivational and ethical leadership [57, 60], continuous professional development [57], interdisciplinary collaboration [54, 59], and the active inclusion of residents and families in care planning [18, 60, 61].

Together, these findings illustrate the complexity of delivering high-quality, ethically grounded care in institutional settings. Residents in nursing homes often present with multiple and interacting vulnerabilities, including chronic illness, functional decline, emotional distress, and social isolation [55, 56, 60], requiring flexible, relationship-based, and person-centred approaches to care.

## Discussion

The findings of this integrative review highlight the significant complexity involved in delivering person-centred care (PCC) to institutionalised older adults with palliative needs. Residents often encounter a number of challenges that accumulate over time, including a loss of autonomy, functional decline, and distress related to identity, dignity, and proximity to end-of-life [55, 56] and are further supported by the broader literature [14, 62, 63]. These difficulties are shaped by a wide range of cultural, spiritual, socioeconomic, and biographical factors [64, 65], which underscore the need for a more personalised and holistic approach.

Person-centred care, as proposed by McCormack and McCance and advanced by the Gothenburg Centre for Person-Centred Care (GPCC), emerges as a relevant framework capable of addressing these multidimensional needs [24, 26]. As demonstrated by the included studies [18, 58, 60] effective communication and relational skills play a pivotal role in personalising care, fostering empathy, and facilitating shared decision-making. These findings are consistent with the broader literature [62–66]. For instance, Saarnio et al. (2017, 2019) emphasised the concept of "at-homeness," which is achieved through respectful environments and trusted relationships that enhance the dignity and well-being of terminally ill residents [55, 56], and is supported by the broader literature [67–75].

Ethical considerations were another key element across the included studies. Holmberg and Godskesen (2022) explored moral sensitivity and relational integrity as essential components for preserving dignity in care [54]. However, only two of the included studies explicitly addressed human rights or ethical principles as core elements [54, 60]. International frameworks, such as those from the WHO and EAPC, emphasise human rights as foundational to palliative care and the protection of dignity in ageing populations [76, 77]. The limited explicit integration of these principles in the included studies may therefore indicate a gap between practice and internationally recognised standards.

Leadership was also identified as a key factor. Bökberg et al. (2019) demonstrated that even in institutions already aligned with PCC principles, the sustained impact of palliative initiatives depended on strong, supportive leadership [57]. However, only one study within this review addressed the inclusion of managers in educational programmes, suggesting an underrepresentation of leadership development in the current literature. Strengthening leadership competencies through structured development programmes, such as mentoring initiatives and organisational policies, could be essential to ensure sustainable change, aligning with OECD (2024) recommendations that prioritise leadership and effective team coordination for system-level transformation [5, 78].

While the included studies [18, 54–58, 60] highlighted the importance of communication and palliative care competencies, only three explicitly recommended structured staff education. This gap is particularly relevant given broader evidence linking training to improved outcomes and reduced hospitalisations [36, 43, 45, 47, 68]. The World Health Organisation (WHO) and the European Association of Palliative Care (EAPC) have recommended integrating basic training in palliative care as a requirement for ensuring quality care and respect for human rights. The discrepancy between the recognised need and the reality of training practices was especially apparent during the course of the pandemic, which exposed systemic vulnerabilities and shortcomings in ethical, communicational and organisational dimensions [45, 79–81].

The findings regarding the integration of fundamental palliative care competencies into routine institutional practice align with both the included studies [60] and the broader literature [82, 83]. This underscores the necessity for fundamental palliative care to be integrated into routine institutional practices, facilitated by interdisciplinary teams and guided by ethical leadership.

Transdisciplinary collaboration emerged as a critical facilitator of high-quality care. In contrast to multidisciplinary approaches, which compartmentalise roles based on discipline, and interdisciplinary approaches, which only facilitate limited integration, transdisciplinary care promotes the collaborative development of solutions that draw from clinical, experiential and contextual knowledge [24, 41].

Evidence from the included studies supports this perspective. Schenell et al. (2020) and Kastbom et al. (2019) reported that models based on self-determination and shared planning are more likely to align with residents' values and improve quality of life [58, 59]. Similarly, Mikaelsson (2025) highlights the value of proactive end-of-life conversations in residential care, showing that early, empathetic communication fosters emotional preparedness, trust, and a sense of control among residents and their families [61]. The study reinforces the need to create structured opportunities for dialogue, not only at the onset of terminal illness, but as part of ongoing, person-centred care planning. These findings are consistent with international recommendations on Advance Care Planning, as advocated by the European Association for Palliative Care (EAPC) [45], reinforcing the importance of embedding structured, ongoing conversations within person-centred care.

**Fig. 2:**
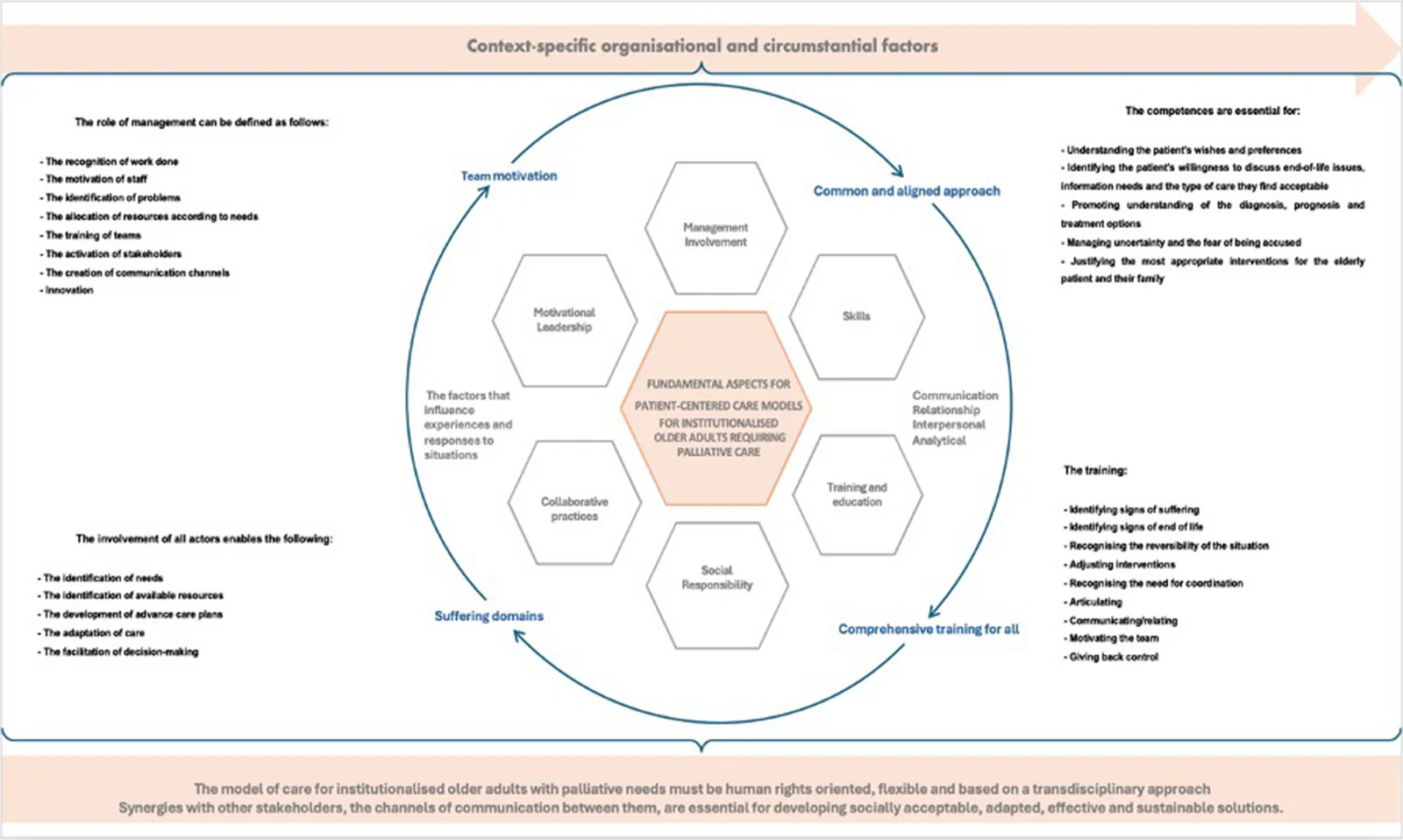
Key elements of a person-centred care model for institutionalised older adults with palliative care needs

To facilitate understanding and practical application of these findings, the essential elements identified across the included studies were synthesised into a visual framework (see Fig. 2). The model is structured around four key dimensions: (1) professional competencies (communication, interpersonal, and analytical skills); (2) involvement of all stakeholders (older adults, families, and healthcare professionals) in the identification of needs, care planning, and decision-making; (3) leadership roles in team coordination and resource management; and (4) guiding principles such as respect for human rights, transdisciplinary collaboration, and social sustainability. As shown in Fig. 2, these dimensions provide a consolidated framework for the ethical and effective delivery of person-centred palliative care in nursing homes. The contextual layer surrounding the model highlights the organisational and circumstantial factors that influence successful implementation, providing an integrated reference framework for delivering ethical, flexible, and sustainable palliative care aligned with the needs and values of institutionalised older adults.

Despite the growing body of evidence supporting PCC and palliative models, significant barriers remain. These include low staff qualifications, time constraints, poor working conditions, and limited institutional support, as identified in the included studies [18, 54, 58–60] and supported by the broader literature [36]. Conversely, key facilitators identified across the included studies include strong leadership, staff motivation, and continuous training strategies [57, 59, 60], which are further reinforced by the broader literature [40, 41]. Addressing these barriers requires not only institutional commitment but also alignment with global frameworks such as those of the WHO and EAPC, ensuring that policies and practices in long-term care are ethically robust, rights-based, and capable of delivering high-quality, dignity-focused care.

To synthesise the essential dimensions identified in this integrative review for the implementation of person-centred care (PCC) in nursing home or institutional settings, a visual framework was developed and is presented in this figure. The model is structured around four interrelated dimensions:

- Professional competencies, including communication, relational, and analytical skills
- Stakeholder involvement, emphasising collaborative planning and shared decision-making between residents, families, and healthcare teams
- Leadership and management, focusing on team coordination, communication channels, and resource allocation
- Core guiding principles, such as social responsibility, human rights, and transdisciplinary collaboration

The contextual layer surrounding the model highlights the organisational and circumstantial factors that influence successful implementation. Together, these elements offer an integrated reference framework for delivering ethical, flexible, and sustainable palliative care aligned with the needs and values of institutionalised older adults. Building on this perspective, recent international research has further reinforced the importance of integrated, collaborative, and ethically grounded care pathways. Hirakawa (2022) emphasised that the implementation of value-based healthcare for older adults requires approaches that integrate dignity, autonomy, and person-centred outcomes. Importantly, the study also reinforces the role of collaborative practices as an essential dimension of high-quality care, resonating with the transdisciplinary and participatory strategies identified in this review [84]. Similarly, the European cross-sectional study on goals-of-care discussions in acute geriatric units highlighted not only the persistent lack of structured communication and treatment limitation decisions across clinical contexts, but also the need to strengthen articulation between hospital services and long-term care providers [85]. This finding underscores the importance of continuity and coordination of care pathways for older adults, ensuring that person-centred and ethically grounded approaches are sustained across settings.

### Study limitations and contributions

The heterogeneity of the included studies, in terms of design, objectives, and participant perspectives, may limit the generalisability of the findings. Furthermore, the preponderance of studies conducted in Sweden may limit the cultural diversity and broader applicability of the findings. Another limitation is the scarcity of studies directly addressing older adults' own perspectives, which represents an important gap in the literature and should be prioritised in future research.

Despite these limitations, this review provides a comprehensive synthesis of strategies, barriers, and facilitators associated with implementing PCC for institutionalised older adults with palliative needs. It emphasises the significance of effective communication, ethical conduct, staff education, and interdisciplinary collaboration all of which are aligned with global healthcare priorities of sustainability, equity, and human rights.

## Conclusion

This integrative review highlights the pressing need for structured, ethically sound, person-centred palliative care in nursing homes, particularly for institutionalised older adults. The synthesis of current evidence demonstrates that approaches grounded in dignity, ethical values, and collaborative planning improve resident experiences and enhance team well-being.

It also emphasises the critical role of interpersonal, communicative, and analytical competencies in ensuring high-quality care, alongside the importance of leadership and ongoing professional development in promoting sustainable, ethical care delivery.

By addressing both structural and relational dimensions, this review contributes to the advancement of geriatric and palliative care knowledge. The findings support policy and practice strategies that enhance quality of life and reinforce the integration of care between nursing homes, hospitals, and community services, ensuring continuity, equity, and value-based and person-centred palliative care for older adults.

## Supplementary Information

Supplementary Material 1: Supplementary File 1 – JBI Critical Appraisal Table. This file presents the assessment of methodological quality of all studies included in the review, conducted using the JBI Critical Appraisal Checklist for Qualitative Research. Supplementary File 2 – PRISMA 2020 Checklist. This file contains the completed PRISMA 2020 checklist, indicating the location within the manuscript where each reporting criterion has been addressed. The checklist confirms adherence to international standards for systematic and integrative reviews. Supplementary File 3 – Supplementary_Materials. This document provides additional methodological details, including eligibility criteria (PICo framework), summary of the search strategy, and synthesized data extraction in tabular form. It also includes a narrative summary of the review process and supporting information relevant to study selection and synthesis. Supplementary File 4 – PRISMA Flow Diagram (Figure 1). A visual representation of the study selection process following PRISMA 2020 guidelines, illustrating the number of records identified, screened, excluded, and included in the final synthesis.

## Data Availability

All data generated or analysed during this study are included in this published article and its supplementary materials. Additional information is available from the corresponding author upon reasonable request.
