# Supplementary material for: Person-centred care models for institutionalised older adults requiring palliative care: an integrative review
Source: BMC Geriatr. 2026 Jul 7;26:1235. doi: 10.1186/s12877-026-07795-y (PMC13628849; doi:10.1186/s12877-026-07795-y)
Supplement: Supplementary file 1 [file 12877_2026_7795_MOESM1_ESM.zip › Material suplementar /Supplementary File 1 ΓÇô JBI Critical Appraisal Table.pdf]

JBI Critical Appraisal Checklist for Qualitative Research

Title:	Autor(a) /Ano	Q1: Is there congruity between the stated philosophical perspective and the research methodology?				Q2: Is there congruity between the research methodology and the research question or objectives?				Q3: Is there congruity between the research methodology and the methods used to collect data?				Q4: Is there congruity between the research methodology and the representation and analysis of data?				Q5: s there congruity between the research methodology and the interpretation of results?				Q6: Is there a statement locating the researcher culturally or theoretically?				Q7: Is the influence of the researcher on the research, and vice- versa, addressed?				Q8: Are participants, and their voices, adequately represented?				Q9: Is the research ethical according to current criteria or, for recent studies, and is there evidence of ethical approval by an appropriate body?				Q10: Do the conclusions drawn in the research report flow from the analysis, or interpretation, of the data?				Overall appraisal:		
		yes	No	Unclear	N/A	yes	No	Unclear	N/A	yes	No	Unclear	N/A	yes	No	Unclear	N/A	yes	No	Unclear	N/A	yes	No	Unclear	N/A	yes	No	Unclear	N/A	yes	No	Unclear	N/A	yes	No	Unclear	N/A	yes	No	Unclear	N/A	Include	Exclude	Seek further info
A qualitative study of nurses' perspective about the impact of end-of-life communication on the goal of end-of-life care in nursing home	(18) Gonella et al. (2021)	x				x				x				x				x					x				x			x				x				x				X		
Barriers to and facilitators of ethical encounters at the end of life in a nursing home: an ethnographic study	(55) Holmberg et al. (2022)	x				x				x				x				x							x		x			x				x				x				X		
Enabling At-Homeness for Older People With Life-Limiting Conditions: A Participant Observation Study From Nursing Homes	(56) Saarnio et al. (2019)			x		x				x				x				x							x		x					x		x				x				x		
Enabling at-homeness for residents living in a nursing home: Reflected experience of nursing home staff	(57) Saarnio et al. (2017)	x				x				x				x				x							x		x			x				x				x				x		
Evaluation of person-centeredness in nursing homes after a palliative care intervention: Pre- and post-test experimental design.	(58) Bökberg et al. (2019)	x				x				x				x				x					x				x			x				x				x				x		
To make and execute decisions throughout life: A person-centred model that facilitates self-determination in residential care, developed through	(59) Schenell et al. (2000)			x		x				x				x				x						x			x				x			x				x				X		
'We have no crystal ball' - advance care planning at nursing homes from the perspective of nurses and physicians	(60) Kastbom et al. (2019)			x			x			x				x				x					x				x			x				x				x				X		
The palliative care experience in Irish nursing homes during the COVID-19 pandemic:a survey of residents, family, and staff	(61) Doody et al.(2024)	x				x				x				x				x					x				x			x				x				x				x		
Proactive end-of-life conversations in residential care homes: a qualitative interview study exploring residents' and family members' experiences	(54) Mikaelsson et al. (2025)	x				x				x				x				x					x				x			x				x				x				x		
