# Supplementary material for: Person-centred care models for institutionalised older adults requiring palliative care: an integrative review
Source: BMC Geriatr. 2026 Jul 7;26:1235. doi: 10.1186/s12877-026-07795-y (PMC13628849; doi:10.1186/s12877-026-07795-y)
Supplement: Supplementary file 1 [file 12877_2026_7795_MOESM1_ESM.zip › Material suplementar /Fluxograma PRISMA.pdf]

PRISMA 2020 flow diagram for new systematic reviews which included searches of databases and registers only

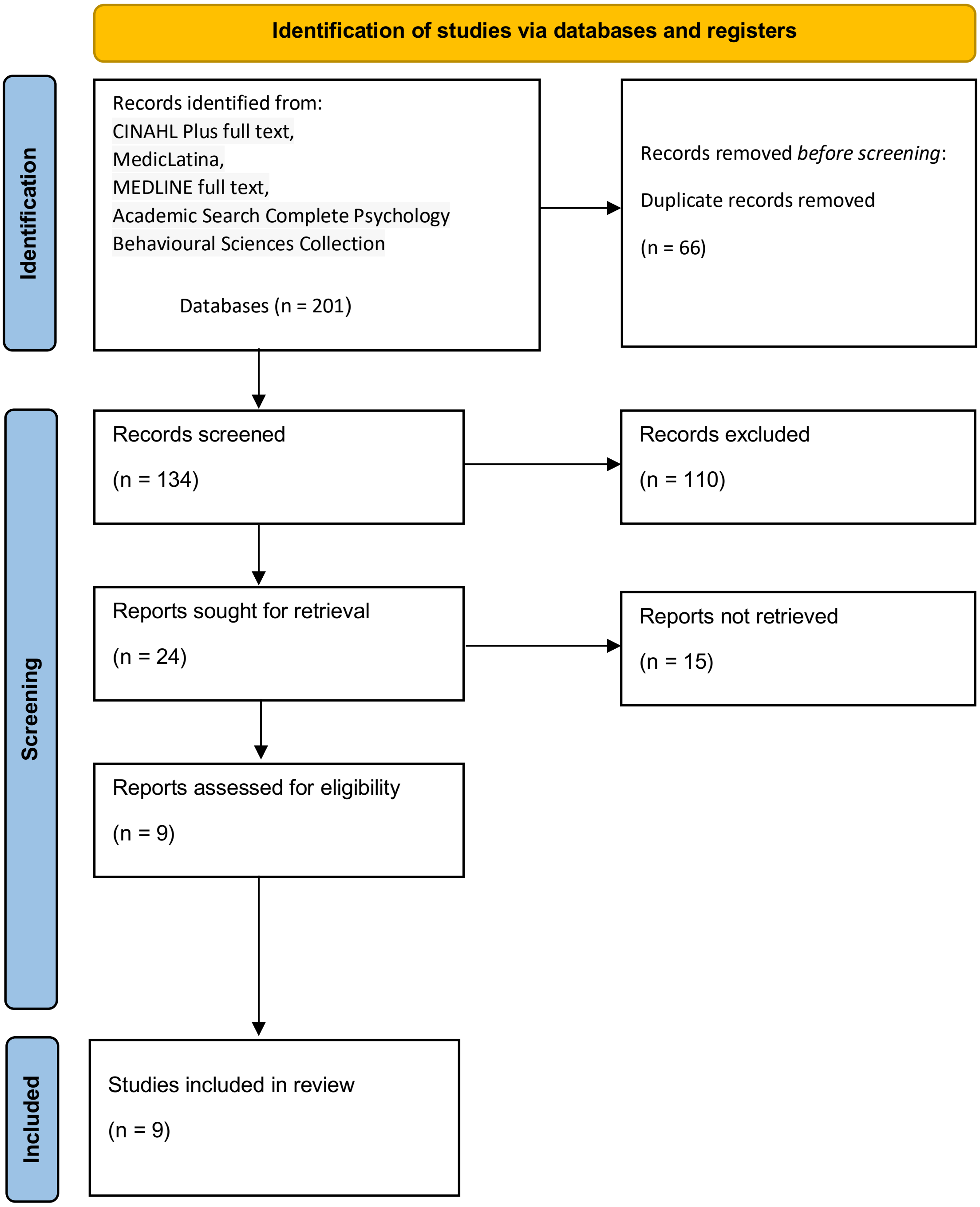

*Consider, if feasible to do so, reporting the number of records identified from each database or register searched (rather than the total number across all databases/registers).

**If automation tools were used, indicate how many records were excluded by a human and how many were excluded by automation tools.

From: Page MJ, McKenzie JE, Bossuyt PM, Boutron I, Hoffmann TC, Mulrow CD, et al. The PRISMA 2020 statement: an updated guideline for reporting systematic reviews. BMJ 2021;372:n71. doi: 10.1136/bmj.n71

For more information, visit: http://www.prisma-statement.org/

PRISMA 2020 flow diagram for new systematic reviews which included searches of databases and registers only
