# Supplementary material for: Person-centred care models for institutionalised older adults requiring palliative care: an integrative review
Source: BMC Geriatr. 2026 Jul 7;26:1235. doi: 10.1186/s12877-026-07795-y (PMC13628849; doi:10.1186/s12877-026-07795-y)
Supplement: Supplementary file 1 [file 12877_2026_7795_MOESM1_ESM.zip › Material suplementar /RIL 24.10.2025 (Corrected).pdf]

Person-Centred Care Models for institutionalised older adults requiring palliative care: An integrative review

Lucinda Marques [1, 2*, [0000-0001-7661-404X]], Bruno Morgado [3,4 [0000-0002-9931-3677]], Alícia Oliveira [5 [0000-0003-0833-0736]], Ana Lazana [5 [0000-0001-5875-4866]], Lara Guedes Pinho [6,2,7 [0000-0003-1174-0744]] Isabel Bico [1,2 [0000-0002-3868-2233]] and Manuel José Lopes [1,2,7 [0000-0002-7554-8041]]

1 Universidade de Évora, Nursing Department 7000-811 Évora, Portugal

2 Comprehensive Health Research Centre (CHRC), 7000-811 Évora, Portugal

3 Universitat Rovira e Virgilli, Department of Nursing, Tarragona, Spain

4 Polytechnic Institute of Portalegre, Portalegre, Portugal

5 Unidade Local de Saúde Alentejo Central, 7000 - 811 Évora, Portugal

6 Escola Superior de Saúde do Instituto Politécnico de Viana do Castelo, Nursing Department 4900-314 Viana do Castelo, Portugal

7 LA REAL - Associated Laboratory in Translation and Innovation Towards Global Health, University of Évora, Portugal

[*] Corresponding author(s). E-mail(s): luenf27@gmail.com, Contributing authors: morgadobruno2@hotmail.com, aliciagsoliveira@gmail.com, analazana@gmail.com, lmgp@uevora.pt, isabelbico@uevora.pt, mjl@uevora.pt

Abstract

Background

This review explores strategies for implementing person-centred care (PCC) for older adults in institutional care settings with palliative care needs. Such settings present complex challenges, including loss of autonomy, multiple chronic conditions and psychological or existential distress. Integrating PCC principles with palliative care is a suitable approach as it promotes shared decision-making, dignity, comfort and well-being. This approach contributes to individual satisfaction and the sustainability of care systems.

Aim

To synthesise the scientific evidence on person-centred care models for institutionalised older adults in need of palliative care.

Materials and methods

A literature review was conducted using five databases. The initial search covered the period from Dec 17 to Aug 22, reflecting palliative and person-centred developments. An additional search was performed between Sep 22 and June 25 to ensure coverage. The search strategy incorporated Medical Subject Headings (Mesh) terms and equivalent descriptors, including palliative care, end-of-life care, palliative nursing, long-term care, nursing homes, residential care, person-centred care, patient-centred care and client-centred care. The review was conducted in accordance with the PRISMA methodology and was registered in the PROSPERO database (CRD42022373388).

Results

Effective implementation of PCC requires more than technical proficiency; it demands advanced communication, interpersonal, reflective and analytical skills, which are rooted in ethical reasoning and collaborative practice. Major barriers included insufficient staff competencies, limited training, resource constraints, and institutional rigidity. Facilitators included motivational leadership, continuous professional development, interdisciplinary collaboration, and institutional social responsibility. These findings are consistent with the Person-Centred Practice Framework developed by McCormack and McCance and the work of the University of Gothenburg's Centre for Person-Centred Care. Both frameworks emphasise co-constructed care planning and respect for patient narratives as core principles of PCC.

Conclusion

This integrative review provides a comprehensive synthesis of the conditions that enable PCC implementation in long-term care. The findings emphasise the importance of ethical care practices,

structural support, and ongoing investment in workforce development. The study reinforces the relevance of transdisciplinary, human rights–based models and offers practical recommendations for care delivery, education, and policy design.

Keywords: person-centred care, palliative care, institutionalised older adults, interdisciplinary collaboration, communication, long-term care, human dignity, healthcare ethics, training, transdisciplinary

Introduction

Technical, scientific, and socio-economic advances over recent decades have led to significant improvements in public health, reflected in increased life expectancy. However, declining birth and mortality rates have been major contributors to population ageing, one of the most significant social changes of the 21st century, with far-reaching implications across all sectors of society (1-3).

The global population aged 60 and over is projected to double from 962 million in 2017 to 2.1 billion by 2050, while the population aged 80 and over is expected to triple (3). According to the OECD, the proportion of individuals aged 65 and over will rise from 18% in 2021 to 27% by 2050. In countries such as Portugal, this proportion will exceed one-third, with more than one in eight people being 80 or older (4). Despite gains in longevity, this trend has not been accompanied by equivalent increases in healthy life expectancy or significant health improvements (5).

The coexistence of chronic illness, functional decline, and caregiver shortages contributes significantly to the institutionalisation of older adults (6), raising major public health concerns due to their complex, multidimensional needs (5, 7-10). Many long-term care residents now present with advanced frailty, multiple chronic conditions, and polypharmacy, complicating clinical management (11-15).

Erving Goffman's concept of the "total institution" (1961) remains central to understanding institutionalization's impact on autonomy and personal identity (16). Despite policy and technological progress, institutional care remains heterogeneous and fragmented, shaped by each country's healthcare system, social care resources, and funding models (17). In this context,

redefining "health needs" becomes essential, especially given their multidimensional nature, encompassing physical, psychological, social, therapeutic, and existential aspects (15, 18-20). Ageing societies demand integrated, sustainable responses. These require complex thinking and transdisciplinary strategies, moving beyond siloed approaches and fostering co-created, evidence-informed solutions (5,21-23).

A holistic paradigm is essential-one that considers not only health and disease but also personal values, beliefs, preferences, and life goals (24,25). Person-centred care (PCC) and palliative care have emerged as complementary models that respond to this challenge, aligning with frameworks such as McCormack and McCance's person-centred practice theory and the University of Gothenburg's Centre for Person-Centred Care (GPCC) (19,20,24,26-28).

Drawing on Carl Rogers' humanistic psychology, PCC emphasises empathy, acceptance, and autonomy throughout the life course (29), while Thomas's transformative model of ageing (1991) introduced a more dynamic and active vision of later life (30). Studies increasingly support the benefits of PCC, especially in populations with multimorbidity and at the end of life by incorporating individual values and preferences into care planning, improving both outcomes and patient experience (19,20,31,32). Supporting this paradigm shift requires healthcare professionals trained not only in technical but also in relational and ethical competencies (19,20). In this context, palliative care plays a critical role, ensuring an integrated and compassionate approach to suffering in all its dimensions - physical, emotional, social, and spiritual (12,33-38). According to the World Health Organization (WHO), palliative care improves quality of life for patients and families through early identification and appropriate management of distressing symptoms, while affirming life and viewing death as a natural process (27,39).

Integrating palliative care into long-term care institutions is increasingly recognised as best practice for managing frailty and chronic disease in ageing populations (11,27,28,38). Beyond addressing symptoms, it fosters dignity, respects patient preferences, and engages family members. However, implementing these changes requires skilled teams and organizational models that support interdisciplinary collaboration (20,28,40-43).

Current evidence indicates that a significant proportion of institutional care is provided by personnel who lack the necessary qualifications, frequently without higher education or specialized training. This situation compromises the quality of care and places strain on health systems (44-47). According to OECD data, less than 25% of long-term care staff possess higher education qualifications, with over 70% working as uncertified caregivers (46). This lack of training contributes to suboptimal practices and increased use of emergency services by institutionalized residents (28,40,48,49).

Structured education in palliative care and PCC is thus essential not only to meet the demands of multimorbidity and ageing but also to promote compassionate, efficient care. International bodies such as the EAPC and WHO have called for the integration of these fields into both undergraduate curricula and continuing professional education (5). Theoretical frameworks like McCormack and McCance's Middle Range Theory and the GPCC model provide strong foundations for implementing PCC through active listening, collaborative care planning, and a focus on dignity and well-being (24,26).

Moreover, discussions on sustainability, equity, and healthcare efficiency underscore the importance of these approaches. Research confirms that palliative and person-centred care reduce avoidable hospitalisations, improve patient and caregiver satisfaction, and support more sustainable healthcare systems (15,35,50-52). Investing in training and implementing these care models is not only an ethical obligation, but also a strategic necessity when addressing today's demographic and clinical challenges.

In view of the increasing number of institutionalised older adults with complex and multidimensional care needs, particularly those related to chronic illness and end-of-life trajectories, there is an urgent need to rethink and strengthen care models within long-term care settings, especially in the context under study. However, current practices remain fragmented, and the evidence on person-centred palliative approaches is limited and heterogeneous. The purpose of this review is to provide a summary of the scientific evidence on care strategies for institutionalised older adults with palliative care needs.

Material e Methods

This study was developed as an integrative review of the literature, with the aim of synthesising empirical evidence on models of person-centred care (PCC) for institutionalised older adults with palliative needs.

Search Strategy

Relevant publications were identified through comprehensive searches in the following full-text electronic databases: CINAHL Plus, MedicLatina, MEDLINE, Academic Search Complete, and Psychology and Behavioural Sciences Collection.

The search strategy combined Medical Subject Headings (MeSH) and equivalent terms related to both *palliative care* and *person-centred approaches*. Key terms included: *palliative care*, *end-of-life care*, *palliative nursing*, *long-term care*, *nursing homes*, *residential care*, *person-centred care*, *patient-centred care*, and *client-centred care*.

The initial search covered studies published between December 2017 and August 2022, a period selected to capture recent developments following global health policy reforms promoting integrated and humanised long-term care. To ensure the review remained current, an updated search was carried out between September 2022 and June 2025.

This review was conducted in accordance with the Preferred Reporting Items for Systematic Reviews and Meta-Analyses (PRISMA 2020) guidelines and has been registered in the International Prospective Register of Systematic Reviews (PROSPERO) (Registration No. CRD42022373388) (33).

Eligibility Criteria

The PICOS framework was applied to guide the inclusion and exclusion process (P = population, I = interventions, C = comparator, O = outcome, S = study design) (43). This framework ensured a methodical, transparent, and replicable selection process. The inclusion and exclusion criteria, detailed according to the PICOS structure, are summarised in Table 1.

The overarching research question guiding this study was:

Which care models or interventions focused on institutionalised older adults recognise, consider, and respond to their palliative care needs?

Inclusion Criteria

Studies were included if they:

- Involved institutionalised older adults aged 60 years or over, living in public or private nursing homes (long or short term).
- Described palliative care interventions or approaches consistent with a palliative philosophy (diagnostic assessment, needs identification, care planning, or symptom relief).
- Adopted a person-centred care perspective, addressing autonomy, shared decision-making, and holistic well-being.

Exclusion Criteria

Studies were excluded if they:

- Were conducted in hospital or primary care settings
- Focused on non-institutionalised populations
- Were systematic reviews, meta-analyses, or theoretical reflections lacking empirical data.

Inclusion Criteria According to PICOS

Element	Description
**P (Population)**	Institutionalised older adults (≥60 years) living in nursing homes
**I (Intervention)**	Palliative care actions or person-centred care interventions tailored to palliative needs
**C (Comparator)**	Not required / Not applicable (most studies were descriptive or exploratory)
**O (Outcomes)**	Relief of suffering, identification of needs, improvement in quality of life, shared decision-making
**S (Study design)**	Primary studies using qualitative, quantitative, or mixed methods (ethnographic, descriptive, observational)

Table 1. Inclusion Criteria According to PICOS Framework

Study selection and data collection

A total of 201 records were initially identified and imported into Mendeley Reference Manager. Following the removal of 66 duplicates, the remaining studies were screened by two independent reviewers based on title and abstract, with the predefined eligibility criteria being applied. Disagreements were resolved by a third reviewer.

Following a thorough review of the literature, nine studies were found to meet all the inclusion criteria. The selection process for the study is outlined in Figure 1, in accordance with the PRISMA 2020 flowchart.

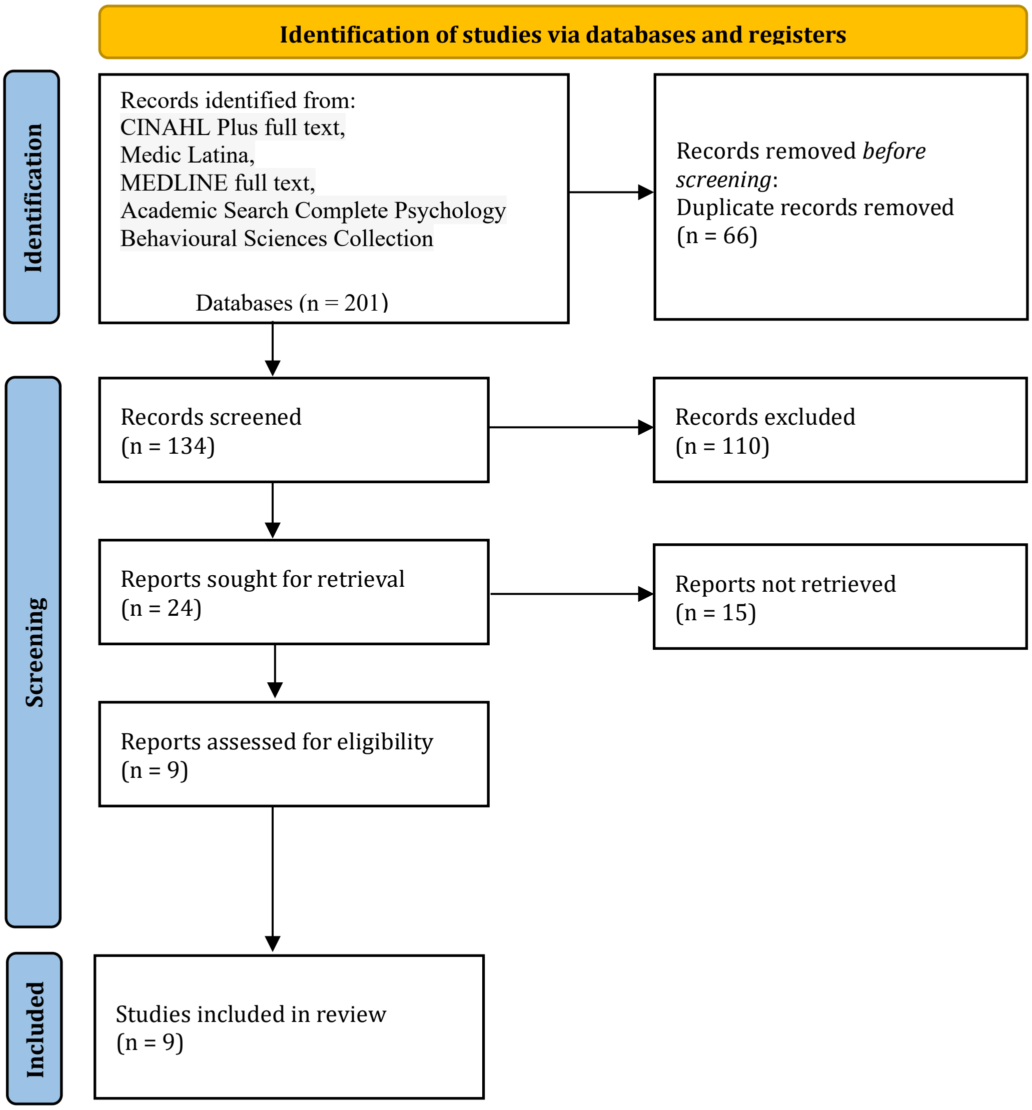

Figure 1. PRISMA 2020 systematic review flowchart of the selection of studies.

Quality Appraisal and Data Analysis

The methodological quality of each study was assessed using the Joanna Briggs Institute (JBI) Critical Appraisal Checklist (53). This tool is designed to evaluate potential bias in study design, conduct, and analysis.

The selected studies were then categorised according to key principles of person-centred and palliative care, including respect for individual values and goals, promotion of autonomy, involvement of families and caregivers in care planning and shared decision-making, holistic and relational care, focus on quality of life, and interdisciplinary collaboration.

Most studies (7 out of 9) highlighted communication and relational skills as being essential for recognising needs and engaging residents and families in decision-making processes. Two studies explicitly discussed ethical principles and human rights, while three proposed structured training programmes for care teams. Only one of the reports recommended the inclusion of managers in educational initiatives, highlighting leadership as a key enabler.

The methodological quality of included studies was assessed using the Joanna Briggs Institute (JBI) Critical Appraisal Checklist for Qualitative Research. This checklist evaluates potential sources of bias in study design, conduct, and analysis. The results are in Supplementary File 1 – JBI Appraisal Table.

Results

The results are presented in two sections: the first provides an overview of the studies included in this integrative review, and the second outlines the key aspects of person-centred care (PCC) as they relate to institutionalised older adults with palliative care needs.

Overview of Included Studies

A total of nine studies were included in the review, all of which were conducted in nursing home settings. Despite variations in methodological approach and context, the studies shared a common aim: to improve or evaluate care models centred on the needs of institutionalised older adults requiring palliative care. Seven of the studies were carried out in Sweden, one in Italy, and one in

Ireland. The settings included both public and private institutions, urban and rural environments, and varied between short- and long-term residential care.

Methodologically, two studies followed a quantitative approach, one of which included a control group, while seven employed qualitative designs, one ethnographic and six descriptive. Four studies explored the perspectives of older residents, two of which also included the views of family members, while the remaining studies focused on healthcare professionals' experiences.

The quality of the included articles was assessed using the Joanna Briggs Institute checklist (53), which evaluates potential bias in study design, conduct, and analysis. Despite methodological heterogeneity, the studies were broadly aligned with the principles of person-centred and palliative care. These include respect for individual values, autonomy, involvement of families and professionals in shared decision-making, and a holistic approach. A detailed summary of the included studies, outlining their aims, designs, methods, and key findings, is presented in Table 2.

Key Aspects of Person-Centred Care

Across the studies, several core principles of person-centred care emerged.

- It is essential to show respect for individual values and care goals
- The promotion of autonomy and self-determination are key components of our business philosophy
- Older adults, families, and caregivers should be involved in shared decision-making
- We offer comprehensive, integrated approaches to care
- Practices that prioritize relationships and a human-centred approach
- We prioritize a consistent focus on quality of life
- The importance of interdisciplinary teamwork in facilitating coordinated care cannot be overstated

It is clear from the seven out of nine studies that communication and relational skills are important for identifying care needs and involving stakeholders in care planning. Notably, the study by Mikaelsson et al. (2025) explored the experiences of residents and their families regarding proactive

end-of-life conversations (54). The findings demonstrated that these early, intentional discussions fostered trust, reduced anxiety, and reinforced the ethical value of shared decision-making in palliative care within residential settings.

Two studies highlighted ethical principles and human rights as fundamental dimensions of care. However, only three studies recommended structured training for staff, and only one study recognized the need to include managers in such initiatives to support effective leadership in palliative care implementation.

Several barriers to the implementation of person-centred palliative care in nursing homes were identified:

- Lack of formal training and qualifications among staff
- Limited involvement of leadership in care processes
- Resource scarcity and inadequate institutional support
- Ethical challenges and communication breakdowns, particularly during crises such as the pandemic

Conversely, the following enablers were recognised:

- Motivational leadership committed to ethical care
- Ongoing opportunities for professional development
- Multidisciplinary collaboration
- Active inclusion of residents and families in care planning and decision-making

The importance of including residents and their families in care planning and decision-making processes was further supported by Mikaelsson et al. (2025), who found that structured conversations not only enhanced participation but also promoted a sense of security and personal control over end-of-life preferences (54).

Taken together, these findings reveal the complexity of delivering high-quality care in institutional settings. Residents frequently exhibit a combination of overlapping vulnerabilities, chronic illnesses,

functional decline, emotional distress, and social isolation. This necessitates the implementation of ethically informed, flexible, and relationship-based care strategies.

Publication and year	Country / Setting	Aim / Focus	Participants / Design	Data collection method	Main Findings (Paraphrased Summary)	Themes / competencies relevant to person-centred care of institutionalised older adults with palliative needs
**(18) Gonella et al. (2021)**	Italy / 13 nursing homes	To examine nurses' experiences and perceptions regarding communication about end-of-life issues with residents and families.	14 nurses/ Qualitative descriptive study	Semi-structured interviews	A few factors affecting end-of-life communication were identified, including the culture of care, staff–resident–family relationships, and the shift from curative to palliative goals. Effective communication is key to improving shared understanding and ensuring that care plans are informed by the preferences of patients and their families.	• Communication skills<br>• Empathy<br>• Shared decision-making<br>• Recognition of preferences
**(55) Holmberg & Godskesen (2022)**	Sweden / Urban nursing home	To explore ethical dimensions and moral sensitivity in encounters between residents and nursing staff during physical care.	12 residents, 16 nursing assistants / Focused ethnography	Participant observation and interviews	Highlighted moral sensitivity and genuine engagement as essential to dignified care. Barriers included lack of resources and communication; facilitators were empathy, dialogue, and ethical reflection.	• Ethical Awareness<br>• Moral Sensitivity<br>• Communication<br>• Reflective Practice
**(56) Saarnio et al. (2019)**	Sweden / Two nursing homes	To identify strategies used by staff to promote a sense of "at-homeness" in residents receiving palliative care.	13 residents, 14 care staff / Interpretive descriptive study	Observations and interviews	The practices identified as key factors as fostering a sense of "at-homeness" include establishing a personal connection with residents, maintaining integrity, fostering family-like relationships, ensuring continuity and encouraging reflective teamwork	• Respect For Integrity<br>• Reliability<br>• Teamwork<br>• Comfort Promotion
**(57) Saarnio et al. (2017)**	Sweden / Non-profit nursing home	To explore how daily routines and relationships create a sense of "at-homeness" for residents in long-term care.	10 healthcare professionals / Interpretive description	Focus group discussions	Emphasised the importance of continuity, personal knowledge of residents, and relational care practices to maintain dignity and identity.	• Continuity<br>• Personalisation<br>• Relational Care<br>• Resident Identity.

**(58) Bökberg et al. (2019)**	Sweden / Nine nursing homes	To evaluate the effects of staff education on perceptions of person-centred palliative care.	365 healthcare staff / Quasi-experimental design	Structured questionnaires (pre and post-test)	At the initial stage of the study, both groups demonstrated high levels of performance. However, after the intervention, there was only a mild improvement that had been measured. Management and organisational support have been identified as the main factors in sustaining PCC.	• Leadership Engagement<br>• Organisational Support<br>• Team Culture.
**(59) Schenell et al. (2020)**	Sweden / Forty residential care facilities	To co-develop a model that promotes self-determination and autonomy in residential care.	Mixed group (staff, residents, managers) / Participatory design	Focus groups and workshops	The "In my way, at my pace, with the help of you" model was produced, with a view to reinforcing relational autonomy through trust, respect and shared decision-making.	• Autonomy<br>• Trust<br>• Empowerment<br>• Collaborative Care.
**(60) Kastbom et al. (2019)**	Sweden / Nine nursing homes	To explore healthcare professionals' experiences with advance care planning (ACP).	25 professionals (physicians, nurses) / Qualitative study	Individual interviews	Four phases of ACP identified: exploring preferences, integrating views, documenting, and revisiting plans. Highlighted the need for collaborative ACP and clear communication.	• Advance Care Planning; Communication<br>• Ethical Discussion.
**(61) Doody et al. (2024)**	Ireland / Twenty-five nursing homes	To examine perceptions of residents, relatives, and staff on palliative and person-centred care provision.	118 participants (residents, family members, staff) / Quantitative cross-sectional study	Structured questionnaires	Reported overall positive experiences but noted communication gaps and resource limitations. Underlined the ethical duty to uphold residents' and professionals' human rights.	• Communication<br>• Human Rights<br>• Ethical Leadership<br>• Staff Education.
**(54) Mikaelsson et al. (2025)**	Sweden / Five residential care homes	To explore residents' and families' experiences of proactive end-of-life conversations.	11 residents, 8 relatives / Qualitative interview study	Semi-structured interviews	Identified benefits such as open dialogue, shared understanding, emotional security, and relationship strengthening. Promoted early discussions as a core element of PCC.	• Open Communication<br>• Trust<br>• Family Involvement<br>• Emotional Preparedness.

Table 2 – Analysis of the selected papers
Summary of the studies included in the integrative review, highlighting methodological characteristics, main findings, and competencies relevant to person-centred care for institutionalised older adults with palliative needs

Discussion

The findings of this integrative review highlight the significant complexity involved in delivering person-centred care (PCC) to institutionalised older adults with palliative needs. Residents often encounter a number of challenges that accumulate over time, including a loss of autonomy, functional decline, and distress related to identity, dignity, and proximity to end-of-life (14,55, 56,62,63). These difficulties are shaped by a wide range of cultural, spiritual, socioeconomic, and biographical factors (64,65), which underscores the need for a more personalised and holistic approach.

Person-centred care, as proposed by McCormack and McCance and advanced by the Gothenburg Centre for Person-Centred Care (GPCC), emerges as a relevant framework capable of addressing these multidimensional needs (24,26). As demonstrated by the findings of numerous studies referenced in this review, effective communication and relational skills play a pivotal role in personalising care, fostering empathy, and facilitating shared decision-making (18,59,61-66). For instance, Saarnio et al. (2017, 2019) emphasised the concept of "at-homeness," which is achieved through respectful environments and trusted relationships that enhance the dignity and well-being of terminally ill residents (55,67,72-75).

Ethical considerations were another key element across the reviewed literature. In their 2022 study, Holmberg and Godskesen examined moral sensitivity and the relational integrity necessary to preserve dignity during care (72,76). However, only two of the eight studies explicitly referenced human rights or ethics as core elements of care, The WHO and EAPC highlight human rights as foundational to palliative approaches and to safeguarding dignity in ageing populations. The lack of explicit integration of these frameworks in institutional practices may therefore reflect an ethical gap and a misalignment with internationally recognised standards. (77)

Leadership was also identified as a key factor. Bökberg et al. (2019) demonstrated that even in institutions already aligned with PCC principles, the sustained impact of palliative initiatives depended on strong, supportive leadership (58). However, only one study in this review addressed the inclusion of managers in educational programmes, suggesting an underrepresentation of leadership development in the current literature. Strengthening leadership competencies through

structured development programmes, such as mentoring initiatives and organisational policies, could be essential to ensure sustainable change, aligning with OECD (2024) recommendations that prioritise leadership and effective team coordination for system-level transformation (5,78).

With regard to training, while most studies acknowledged the need for communication and palliative competencies, only three explicitly recommended structured staff education, despite evidence linking training with better outcomes and reduced hospitalisations (18,36,43,45,47,55-59,61,72). The World Health Organisation (WHO) and the European Association of Palliative Care (EAPC) have recommended integrating basic training in palliative care as a requirement for ensuring quality care and respect for human rights. The discrepancy between the recognised need and the reality of training practices was especially apparent during the course of the pandemic, which exposed systemic vulnerabilities and shortcomings in ethical, communicational and organisational dimensions (45,79-81).

The findings also align with the idea that while some situations require specialised palliative intervention, many can be effectively managed by generalist staff equipped with foundational training (61,82,83). This underscores the necessity for fundamental palliative care to be integrated into routine institutional practices, facilitated by interdisciplinary teams and guided by ethical leadership.

Transdisciplinary collaboration emerged as a critical facilitator of high-quality care. In contrast to multidisciplinary approaches, which compartmentalise roles based on discipline, and interdisciplinary approaches, which only facilitate limited integration, transdisciplinary care promotes the collaborative development of solutions that draw from clinical, experiential and contextual knowledge (24,41). Schenell et al. (2020) and Kastbom et al. (2019) both support this perspective, noting that models based on self-determination and shared planning are more likely to align with residents' values and improve quality of life (36,59). Similarly, Mikaelsson (2025) highlights the value of proactive end-of-life conversations in residential care, showing that early, empathetic communication fosters emotional preparedness trust and a sense of control among residents and their families (54). The study reinforces the need to create structured opportunities for dialogue, not only at the onset of terminal illness, but as part of ongoing, person-centred care

planning (9). These findings are consistent with international recommendations on Advance Care Planning, as advocated by the EAPC, reinforcing the importance of embedding structured, ongoing conversations within person-centred care.

To facilitate understanding and practical application of these findings, the essential elements of a proposed person-centred care model for institutionalised older adults have been synthesised into a visual framework (see Figure 2). The model is structured around four key dimensions: (1) professional competencies (communication, interpersonal, and analytical skills); (2) involvement of all stakeholders (older adults, families, and healthcare professionals) in the identification of needs, care planning, and decision-making; (3) leadership roles in team coordination and resource management; and (4) guiding principles such as respect for human rights, transdisciplinary collaboration, and social sustainability. As shown in Figure 2, these dimensions provide a consolidated framework for the ethical and effective delivery of person-centred palliative care in nursing homes. The contextual layer surrounding the model highlights the organisational and circumstantial factors that influence successful implementation, providing an integrated reference framework for delivering ethical, flexible, and sustainable palliative care aligned with the needs and values of institutionalised older adults.

Despite the growing body of evidence supporting PCC and palliative models, significant barriers remain. These include low staff qualifications, time constraints, poor working conditions, and limited institutional support (18,36,55,59-61). Conversely, key facilitators include strong leadership, staff motivation, and the implementation of continuous training strategies (40,41,58,60,61). Addressing these barriers requires not only institutional commitment but also alignment with global frameworks such as those of the WHO and EAPC, ensuring that policies and practices in long-term care are ethically robust, rights-based, and capable of delivering high-quality, dignity-focused care.

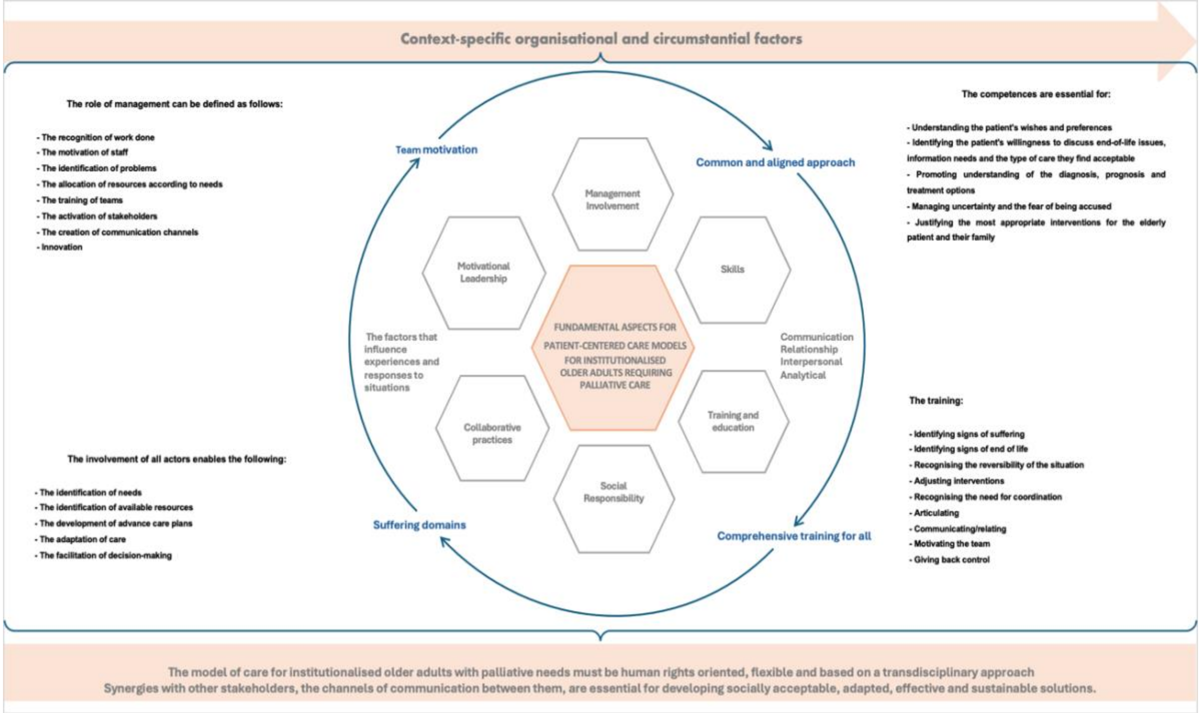

Figure 2. Key elements of a person-centred care model for institutionalised older adults with palliative care needs.

To synthesise the essential dimensions identified in this integrative review for the implementation of person-centred care (PCC) in nursing home or institutional settings, a visual framework was developed and is presented in this figure. The model is structured around four interrelated dimensions:

- **Professional competencies**, including communication, relational, and analytical skills
- **Stakeholder involvement**, emphasising collaborative planning and shared decision-making between residents, families, and healthcare teams
- **Leadership and management**, focusing on team coordination, communication channels, and resource allocation
- **Core guiding principles**, such as social responsibility, human rights, and transdisciplinary collaboration

The contextual layer surrounding the model highlights the organisational and circumstantial factors that influence successful implementation. Together, these elements offer an integrated reference framework for delivering ethical, flexible, sustainable palliative care aligned with the needs and

values of institutionalised older adults. Building on this perspective, recent international research has further reinforced the importance of integrated, collaborative, and ethically grounded care pathways. Hirakawa (2024) emphasised that the implementation of value-based healthcare for older adults requires approaches that integrate dignity, autonomy, and person-centred outcomes. Importantly, the study also reinforces the role of collaborative practices as an essential dimension of high-quality care, resonating with the transdisciplinary and participatory strategies identified in this review (84). Similarly, the European cross-sectional study on goals-of-care discussions in acute geriatric units highlighted not only the persistent lack of structured communication and treatment limitation decisions across clinical contexts, but also the need to strengthen articulation between hospital services and long-term care providers (85). This finding underscores the importance of continuity and coordination of care pathways for older adults, ensuring that person-centred and ethically grounded approaches are sustained across settings.

Study Limitations and Contributions

It is important to acknowledge the limitations of this study. However, the heterogeneity of the studies included, in terms of design, objectives and participant perspectives, limits the generalisability of the findings. Furthermore, the predominance of studies conducted in Sweden may limit the cultural diversity and broader applicability of the evidence. A further limitation is the relative scarcity of studies that directly explore the perspectives of older adults themselves. This is an important gap that future research should seek to address.

Despite these limitations, this review makes a meaningful contribution to the existing body of knowledge. It provides a comprehensive synthesis of strategies, barriers, and facilitators associated with the implementation of person-centred care (PCC) for institutionalised older adults with palliative needs. The findings emphasise the importance of effective communication, ethical practice, staff education, and interdisciplinary and transdisciplinary collaboration. These elements are strongly aligned with contemporary healthcare priorities, including sustainability, equity, and the protection of human rights. By identifying key competencies and organisational enablers, this review offers a solid foundation to inform future research, guide practice improvements, and support policy development.

Conclusion

This integrative review underlines the urgent requirement for a structured, ethically sound, person-centred approach to palliative care in nursing homes, especially for institutionalised older adults in need of such care. The synthesis of current evidence demonstrates that person-centred approaches rooted in dignity, ethical values, and collaborative planning contribute to improved resident experiences and better working conditions for care teams.

This work highlights the pivotal role of interpersonal, communicative, and analytical competencies in ensuring the delivery of high-quality care. Furthermore, it emphasises the importance of robust leadership and continuous professional development in enabling ethical, effective and sustainable care delivery.

In summary, this review contributes to the academic discourse on geriatric and palliative care, offering practical insights for long-term care institutions and policymakers. By addressing both structural and relational dimensions of care, it supports strategies aimed at enhancing the quality of life for institutionalised older adults with palliative needs. These contributions, further reinforced by recent international research, underscore the importance of enhancing the coordination between nursing homes, hospital services, and community care. Such integration is essential to ensure continuity, equity, and the delivery of value-based, person-centred palliative care across all care settings for older people.

Supplementary Materials

The following supplementary files are provided with this manuscript to ensure transparency, reproducibility, and alignment with the PRISMA 2020 reporting standards.

- **Supplementary File 1 – JBI Critical Appraisal Table**

This file presents the assessment of methodological quality of all studies included in the review, conducted using the JBI Critical Appraisal Checklist for Qualitative Research.

- **Supplementary File 2 – PRISMA 2020 Checklist**

This file contains the completed PRISMA 2020 checklist, indicating the location within the manuscript where each reporting criterion has been addressed. The checklist confirms adherence to international standards for systematic and integrative reviews.

- **Supplementary File 3 – Supplementary_Materials_BMC.docx**

This document provides additional methodological details, including eligibility criteria (PICOS framework), summary of the search strategy, and synthesized data extraction in tabular form. It also includes a narrative summary of the review process and supporting information relevant to study selection and synthesis.

- **Supplementary File 4 – PRISMA Flow Diagram (Figure 1)**

A visual representation of the study selection process following PRISMA 2020 guidelines, illustrating the number of records identified, screened, excluded, and included in the final synthesis.

Availability of Data and Materials

All data generated or analysed during this study are included in this published article and its supplementary materials. Additional information is available from the corresponding author upon reasonable request.

Acknowledgements

The authors acknowledge the University of Évora for institutional support related to the publication of this article.

Funding

The authors declare that this research did not receive any specific grant from funding agencies in the public, commercial, or not-for-profit sectors. Publication of this article was supported under the University of Évora’s institutional protocol.

Competing interests

The authors declare that they have no competing interests.

Trial registration

Not applicable.

Ethics approval and consent to participate

Not applicable.

Consent for publication

Not applicable.

Reference

1. Maia LC, de Figueiredo Braga Colares T, de Moraes EN, de Melo Costa S, Caldeira AP. Robust older adults in primary care: Factors associated with successful aging. Rev Saude Publica. 2020;54.
2. Azevedo L, Riscado P, Maia C. The Influence of Active Aging In The Quality Of Life Of The Elderly-Integrative Literature Review. 2022 Jun;17–27.
3. United Nations. Envelhecimento [Internet]. 2024 [cited 2024 Apr 28]. Available from: https://unric.org/pt/envelhecimento/

4. Health at a Glance 2023: OECD Indicators [Internet]. OECD; 2023 Nov. (Health at a Glance). Available from:https://www.oecd-ilibrary.org/social-issues-migration-health/health-at-a-glance-2023_7a7afb35-en
5. Health at a Glance: Europe 2024 [Internet]. OECD; 2024. (Health at a Glance: Europe). Available from: https: www.oecd.org/en/publications/health-at-a-glance-europe-2024_b3704e14-en.html
6. Lopes M. Desafios de Inovação em Saúde: Repensar os Modelos de Cuidados. Évora: Coleção Claustro; 2021.
7. Quinaz Romana G, Kislaya I, Salvador MR, Cunha Gonçalves S, Nunes B, Dias C. Multimorbidity in Portugal: Results from the first national health examination survey. Acta Med Port. 2019 Jan 1;32(1):30–7.
8. Beil M, Flaatten H, Guidet B, Sviri S, Jung C, de Lange D, et al. The management of multi-morbidity in elderly patients: Ready yet for precision medicine in intensive care? Crit Care. 2021 Dec 1;25(1).
9. Baré M, Herranz S, Roso-Llorach A, Jordana R, Violán C, Lleal M, et al. Multimorbidity patterns of chronic conditions and geriatric syndromes in older patients from the MoPIM multicentre cohort study. BMJ Open. 2021 Nov 15;11(11).
10. Lopes MJ, de Pinho LG, Fonseca C, Goes M, Oliveira H, Garcia-Alonso J, et al. Functioning and cognition of portuguese older adults attending in residential homes and day centers: A comparative study. Int J Environ Res Public Health. 2021 Jul 1;18(13).
11. Tortajada S, Giménez-Campos MS, Villar-López J, Faubel-Cava R, Donat-Castelló L, Valdivieso-Martínez B, et al. Case management for patients with complex multimorbidity: Development and validation of a coordinated intervention between primary and hospital care. Int J Integr Care. 2017 Apr 1;17(2).
12. Hermans K, Cohen J, Spruytte N, Van Audenhove C, Declercq A. Palliative care needs and symptoms of nursing home residents with and without dementia: A cross-sectional study. Geriatr Gerontol Int. 2017 Oct 1;17(10):1501–7.
13. Morin L, Aubry R, Frova L, MacLeod R, Wilson DM, Loucka M, et al. Estimating the need for palliative care at the population level: A cross-national study in 12 countries. Palliat Med. 2017 Jun 1;31(6):526–36.
14. Fonseca C, de Pinho LG, Lopes MJ, Marques M do C, Garcia-Alonso J. The Elderly Nursing Core Set and the cognition of Portuguese older adults: a cross-sectional study. BMC Nurs. 2021 Dec 1;20(1).

15. de Freitas GC, Das Flores JA, de Camargo KR. "Health needs:" reflections on the (in)definition of a concept. Saude e Sociedade. 2022;31(1).

16. Erving Goffman. Asylums: Essays on the social situation of mental patients and other inmates. New York,: Routledge; 2017.

17. Ayalon L, Bengoa R, Cho B, & Comas-Herrera A. Launch of the WHO Global Network on Long-Term Care. 2020 Sep.

18. Gonella S, Basso I, Clari M, Dimonte V, Di Giulio P. A qualitative study of nurses' perspective about the impact of end-of-life communication on the goal of end-of-life care in nursing home. Scand J Caring Sci. 2021 Jun 1;35(2):502–11.

19. Berntsen GKR, Dalbakk M, Hurley JS, Bergmo T, Solbakken B, Spansvoll L, et al. Person-centred, integrated and pro-active care for multi-morbid elderly with advanced care needs: A propensity score-matched controlled trial. BMC Health Serv Res. 2019 Oct 3;19(1).

20. Nkhoma KB, Cook A, Giusti A, Farrant L, Petrus R, Petersen I, et al. A systematic review of impact of person-centred interventions for serious physical illness in terms of outcomes and costs. Vol. 12, BMJ Open. BMJ Publishing Group; 2022.

21. Lopes M. Ciências Da Saúde e Bem-Estar: Contributos Para Uma Perspetiva Epistemológica Integrada. 1a. Castelo Branco : RVJ- Editores, Lda. ; 2024.

22. Antonsen L. What complexity science can teach us about illness and organisation. Tidsskrift para Den norske legeforening. 2019 Jan 25;2:139.

23. Morin E, Lisboa E. Introdução ao Pensamento Complexo . 1o. Gomes luís, editor. Vol. 3. Porto Alegre: Sulina; 2007. 9–118 p.

24. Centre for Person-centred Care - GPCC [Internet]. 2025 [cited 2025 Jun 10]. Available from: https://www.gu.se/en/gpcc/about-our-research?utm_source=chatgpt.com.

25. McCormack B, Dewing J, McCance T. Developing person-centred care: addressing contextual challenges through practice development. Online J Issues Nurs. 2011;16(2):3.

26. McCance T, McCormack B. The Person-centred Nursing Framework: a mid-range theory for nursing practice. Journal of Research in Nursing. 2025 Feb 1;

27. OECD Health Policy Studies. OECD Publishing, Paris. 2023 [cited 2024 Mar 24]. p. 1–243 Time for Better Care at the End of Life. Available from: https://doi.org/10.1787/722b927a-en.

28. Hunter P V., McCleary L, Qiao T, Sussman T, Venturato L, Thompson G, et al. Delivering person-centred palliative care in long-term care settings: is humanism a quality of health-care employees or their organisations? Ageing Soc. 2022 Jun 9;1–18.

29. Rogers C. Tornar-se pessoa . 7. a. Porto: Moraes Editores ; 1985.

30. Thomas WH. Life Worth Living: How someone you love can still enjoy life in a nursing home: The Eden Alternative in action. . VanderWyk&Burnham.; 1996.

31. Ekman I, Swedberg K, Taft C, Lindseth A, Norberg A, Brink E, et al. Person-centered care - Ready for prime time. European Journal of Cardiovascular Nursing. 2011 Dec;10(4):248–51.

32. Wolf A, Ekman I, Dellenborg L. Everyday practices at the medical ward: A 16-month ethnographic field study. BMC Health Serv Res. 2012;12(1).

33. Marques L, Oliveira A, Lazana A, Morgado B, Lopes M, Lara P. Patient-Centered Care Models for Institutionalized Older Adults Requiring Palliative Care: a Systematic Review Protocol. In: Moguel E, Pinho L, Fonseca C, editors. Gerontechnology V: Contributions to the Fifth International Workshop on Gerontechnology, IWoG 2022,. Springer Nature. Springer Nature Switzerland.: Springer Nature; 2022. p. 421–6.

34. Österlind J, Henoch I. The 6S-model for person-centred palliative care: A theoretical framework. Nursing Philosophy. 2021 Apr 1;22(2).

35. Puente-Fernández D, Campos-Calderón CP, Esteban-Burgos AA, Hueso-Montoro C, Roldán-López CB, Montoya-Juárez R. Palliative care symptoms, outcomes, and interventions for chronic advanced patients in Spanish nursing homes with and without dementia. Int J Environ Res Public Health. 2020 Mar 1;17(5).

36. Schenell R, Strang S, Henoch I, Ozanne A. Struggling for a Dignified Life: The Meaning of Self-Determination in Palliative Phase in Residential Care. Int J Hum Caring. 2020 Jun 1;24(2):147–57.

37. Puente-fernández D, Roldán-lópez CB, Campos-calderón CP, Hueso-montoro C, García-caro MP, Montoya-juarez R. Prospective evaluation of intensity of symptoms, therapeutic procedures and treatment in palliative care patients in nursing homes. J Clin Med. 2020 Mar 1;9(3).

38. Mota-Romero E, Tallón-Martín B, García-Ruiz MP, Puente-Fernandez D, García-Caro MP, Montoya-Juarez R. Frailty, complexity, and priorities in the use of advanced palliative care resources in nursing homes. Medicina (Lithuania). 2021 Jan 1;57(1):1–15.

39. Arias-Casais N (Natalia), Garralda E (Eduardo), Rhee JY (John Y), Lima L (Liliana) de, Pons-Izquierdo JJ (Juan J, Clark D (David), et al. EAPC Atlas of Palliative Care in Europe 2019. 2019 [cited 2024 Jan 5]; Available from: https://dadun.unav.edu/handle/10171/56787
40. Whittaker E, George Kernohan W, Hasson F, Howard V, McLaughlin D. The palliative care education needs of nursing home staff. Nurse Educ Today. 2006 Aug;26(6):501–10.
41. Vellani S, Maradiaga Rivas V, Nicula M, Lucchese S, Kruizinga J, Sussman T, et al. Palliative Approach to Care Education for Multidisciplinary Staff of Long-Term Care Homes: A Pretest Post-Test Study. Gerontol Geriatr Med. 2023 Jan 1;9.
42. Outi kiljunen O. Care Home Nursing Professionals' Competence in Older People Nursing [Dissertations in Health Sciences 492]. [Finland]: Department of Nursing Science; 2019.
43. Maeda A, Socha-Dietrich K. Skills for the future health workforce: Preparing health professionals for people-centred care. OECD Health Working Papers [Internet]. 2021;(124). Available from: https://dx.doi.org/10.1787/68fb5f08-en
44. Collingridge Moore D, Payne S, Van den Block L, Ling J, Froggatt K, Gatsolaeva Y, et al. Strategies for the implementation of palliative care education and organizational interventions in long-term care facilities: A scoping review. Vol. 34, Palliative Medicine. SAGE Publications Ltd; 2020. p. 558–70.
45. Payne S, Harding A, Williams T, Ling J, Ostgathe C. Revised recommendations on standards and norms for palliative care in Europe from the European Association for Palliative Care (EAPC): A Delphi study. Palliat Med. 2022 Apr 1;36(4):680–97.
46. Hewlett E, Moran V. Making Mental Health Count [Internet]. OECD; 2014 Jul. (OECD Health Policy Studies). Available from:
https://www.oecd.org/en/publications/making-mental-health-count_9789264208445-en.html
47. Zeng Y, Hu X, Li Y, Zhen X, Gu Y, Sun X, et al. The quality of caregivers for the elderly in long-term care institutions in Zhejiang province, China. Int J Environ Res Public Health. 2019 Jun 2;16(12).
48. Neto I. Pinto J. Pinto HRNI. Envelhecimento: Dimensões e Contextos. In Cuidados paliativos. In Lisboa : Universidade Católica; 2021. p. 195–208.
49. Michielsen L, Bischoff EWMA, Schermer T, Laurant M. Primary healthcare competencies needed in the management of person-centred integrated care for chronic illness and multimorbidity: Results of a scoping review. BMC Primary Care. 2023 Dec 1;24(1).

50. Brereton L, Clark J, Ingleton C, Gardiner C, Preston L, Ryan T, et al. What do we know about different models of providing palliative care? Findings from a systematic review of reviews. Vol. 31, Palliative Medicine. SAGE Publications Ltd; 2017. p. 781–97.

51. Brownie S, Nancarrow S. Effects of person-centered care on residents and staff in aged-care facilities: A systematic review. Vol. 8, Clinical Interventions in Aging. 2013. p. 1–10.

52. Ebrahimi Z, Patel H, Wijk H, Ekman I, Olaya-Contreras P. A systematic review on implementation of person-centered care interventions for older people in out-of-hospital settings. Geriatr Nurs (Minneap). 2021 Jan 1;42(1):213–24.

53. Joanna Briggs Institute [Internet]. Checklist for Systematic Reviews and Research Syntheses. Available from: https://jbi.global/sites/default/files/2019-05/JBI_Critical_Appraisal-Checklist_for_Systematic_Reviews2017_0.pdf

54. Mikaelsson Å, Eriksson LE, Stenfors T, Goliath I. Proactive end-of-life conversations in residential care homes: a qualitative interview study exploring residents' and family members' experiences. BMC Geriatr. 2025 Dec 1;25(1).

55. Holmberg B, Godskesen T. Barriers to and facilitators of ethical encounters at the end of life in a nursing home: an ethnographic study. BMC Palliat Care. 2022 Dec 1;21(1).

56. Saarnio L, Boström AM, Hedman R, Gustavsson P, Öhlén J. Enabling At-Homeness for Older People With Life-Limiting Conditions: A Participant Observation Study From Nursing Homes. Glob Qual Nurs Res. 2019;6.

57. Saarnio L, Boström AM, Hedman R, Gustavsson P, Öhlén J. Enabling at-homeness for residents living in a nursing home: Reflected experience of nursing home staff. J Aging Stud. 2017 Dec 1;43:40–5.

58. Bökberg C, Behm L, Wallerstedt B, Ahlström G. Evaluation of person-centeredness in nursing homes after a palliative care intervention: Pre- and post-test experimental design. BMC Palliat Care. 2019 May 31;18(1).

59. Schenell R, Ozanne A, Strang S, Henoch I. To make and execute decisions throughout life: A person-centred model that facilitates self-determination in residential care, developed through participatory research. Applied Nursing Research. 2020 Oct 1;55.

60. Kastbom L, Milberg A, Karlsson M. 'We have no crystal ball' - advance care planning at nursing homes from the perspective of nurses and physicians. Scand J Prim Health Care. 2019 Apr 3;37(2):191–9.

61. Doody O, Lombard J, Delamere T, Rabbitte M. The palliative care experience in Irish nursing homes during the COVID-19 pandemic: a survey of residents, family, and staff. BMC Palliat Care. 2024 Dec 1;23(1).

62. Barreto JB. Ética nos cuidados a pessoas idosas. Revista Portuguesa de Clínica Geral [Internet]. 2022 Feb 1;38(1):114–24. Available from: https://www.rpmgf.pt/ojs/index.php/rpmgf/article/view/13398

63. Martí-García C, Fernández-Férez A, Fernández-Sola C, Pérez-Rodríguez R, Esteban-Burgos AA, Hernández-Padilla JM, et al. Patients' experiences and perceptions of dignity in end-of-life care in emergency departments: A qualitative study. J Adv Nurs. 2023 Jan 1;79(1):269–80.

64. Clancy A, Simonsen N, Lind J, Liveng A, Johannessen A. The meaning of dignity for older adults: A meta-synthesis. Nurs Ethics. 2021 Sep 1;28(6):878–94.

65. Kaftanski W, Hanson J. Suffering, authenticity, and meaning in life: Toward an integrated conceptualization of well-being. Front Psychol. 2022 Dec 6;13.

66. Kwame A, Petrucka PM. A literature-based study of patient-centered care and communication in nurse-patient interactions: barriers, facilitators, and the way forward. Vol. 20, BMC Nursing. BioMed Central Ltd; 2021.

67. Grover SM. Continuing Education Shaping Effective Communication Skills and Therapeutic Relationships at Work The Foundation of Collaboration. 2005 Apr;

68. Jiang Y, Shi L, Cao J, Zhu L, Sha Y, Li T, et al. Effectiveness of clinical scenario dramas to teach doctor-patient relationship and communication skills. BMC Med Educ. 2020 Dec 1;20(1).

69. Kmetec S, Fekonja Z, Kolarič JČ, Reljić NM, McCormack B, Sigurðardóttir ÁK, et al. Components for providing person-centred palliative healthcare: An umbrella review. Vol. 125, International Journal of Nursing Studies. Elsevier Ltd; 2022.

70. Laranjeira C. Dignity promotion in people with advanced chronic diseases: contributions for a value-based healthcare practice. Front Public Health. 2023;11.

71. Franco ME, Salvetti MDG, Donato SCT, Carvalho RT De, Franck EM. Perception of dignity of patients in palliative care. Texto e Contexto Enfermagem. 2019;28.

72. Muldrew DHL, McLaughlin D, Brazil K. Ethical issues experienced during palliative care provision in nursing homes. Nurs Ethics. 2019 Sep 1;26(6):1848–60.

73. Ryan T. Facilitators of person and relationship-centred care in nursing. Nurs Open. 2022 Mar 1;9(2):892–9.

74. Ferreira M, Martinsone B, Talić S. Promoting Sustainable Social Emotional Learning at School through Relationship-Centered Learning Environment, Teaching Methods and Formative Assessment. Journal of Teacher Education for Sustainability. 2020 Jun 1;22(1):21–36.

75. Baarck Julia, Kovačić Matija. The relationship between loneliness and health. European Union. Luxembourg: Publications Office of the European Union; 2022.

76. Corradi-Perini C, Beltrão JR, Ribeiro URV de CO. Circumstances Related to Moral Distress in Palliative Care: An Integrative Review. Vol. 38, American Journal of Hospice and Palliative Medicine. SAGE Publications Inc.; 2021. p. 1391–7.

77. Perez B, Eler K. Direitos Humanos do paciente idoso no contexto dos cuidados paliativos. Rev Iberoam Bioet. 2024 Oct 31;(26):01–17.

78. Beyond Applause? Improving Working Conditions in Long-Term Care [Internet]. OECD; 2023. Available from: https://www.oecd.org/en/publications/beyond-applause-improving-working-conditions-in-long-term-care_27d33ab3-en.html

79. Evaluación del desarrollo de los cuidados paliativos en el mundo: conjunto de indicadores factibles. Ginebra: Organización Mundial de la Salud; 2022. Licencia: CC BY-NC-SA 3.0 IGO. [Internet]. Ginebra; 2022 [cited 2025 Aug 27]. Available from: https://www.who.int/es/publications/i/item/9789240033351

80. Integrating palliative care and symptom relief into primary health care: a WHO guide for planners, implementers and managers. Geneva: World Health Organization; 2018. Licence: CC BY-NC-SA 3.0 IGO. [Internet]. 2018 [cited 2025 Aug 27]. Report No.: 1–88. Available from: https://www.who.int/publications/i/item/integrating-palliative-care-and-symptom-relief-into-primary-health-care

81. Froggatt KA, Moore DC, Van den Block L, Ling J, Payne SA, Arrue B, et al. Palliative Care Implementation in Long-Term Care Facilities: European Association for Palliative Care White Paper. J Am Med Dir Assoc. 2020 Aug 1;21(8):1051–7.

82. Pazes C, Galvão C, Neto I, Marques L. Acções Paliativas em ERPIs_ uma aposta imprescindível. Ordem dos Médicos [Internet]. 2020 [cited 2024 Jul 5]; Available from: https://ordemdosmedicos.pt/accoes-paliativas-em-erpis-uma-aposta-imprescindivel/

83. Hökkä M, Martins Pereira S, Pölkki T, Kyngäs H, Hernández-Marrero P. Nursing competencies across different levels of palliative care provision: A systematic integrative review with thematic synthesis. Vol. 34, Palliative Medicine. SAGE Publications Ltd; 2020. p. 851–70.

84. Hirakawa Y. Defining and Implementing Value-Based Healthcare for Older People from a Geriatric and Gerontological Perspective. Vol. 19, International Journal of Environmental Research and Public Health. MDPI; 2022.

85. Piers R, Pautex S, Rexach Cano L, Leners JC, Vali Ahmed M, De Brauwer I, et al. Goals of care discussions and treatment limitation decisions in European acute geriatric units: a one-day cross-sectional study. Age Ageing. 2025 Feb 1;54(2).
