# Supplementary material for: Person-centred care models for institutionalised older adults requiring palliative care: an integrative review
Source: BMC Geriatr. 2026 Jul 7;26:1235. doi: 10.1186/s12877-026-07795-y (PMC13628849; doi:10.1186/s12877-026-07795-y)
Supplement: Supplementary file 1 [file 12877_2026_7795_MOESM1_ESM.zip › Material suplementar /Table 2 ΓÇô Analysis of the selected paper .pdf]

Publication and year	Country / Setting	Aim / Focus	Participants / Design	Data collection method	Main Findings (Paraphrased Summary)	Themes / competencies relevant to person-centred care of institutionalised older adults with palliative needs
**(18) Gonella et al. (2021)**	Italy / 13 nursing homes	To examine nurses' experiences and perceptions regarding communication about end-of-life issues with residents and families.	14 nurses/ Qualitative descriptive study	Semi-structured interviews	A few factors affecting end-of-life communication were identified, including the culture of care, staff–resident–family relationships, and the shift from curative to palliative goals. Effective communication is key to improving shared understanding and ensuring that care plans are informed by the preferences of patients and their families.	• Communication skills<br>• Empathy<br>• Shared decision-making<br>• Recognition of preferences
**(55) Holmberg & Godskesen (2022)**	Sweden / Urban nursing home	To explore ethical dimensions and moral sensitivity in encounters between residents and nursing staff during physical care.	12 residents, 16 nursing assistants / Focused ethnography	Participant observation and interviews	Highlighted moral sensitivity and genuine engagement as essential to dignified care. Barriers included lack of resources and communication; facilitators were empathy, dialogue, and ethical reflection.	• Ethical Awareness<br>• Moral Sensitivity<br>• Communication<br>• Reflective Practice
**(56) Saarnio et al. (2019)**	Sweden / Two nursing homes	To identify strategies used by staff to promote a sense of "at-homeness" in residents receiving palliative care.	13 residents, 14 care staff / Interpretive descriptive study	Observations and interviews	The practices identified as key factors as fostering a sense of "at-homeness" include establishing a personal connection with residents, maintaining integrity, fostering family-like relationships, ensuring continuity and encouraging reflective teamwork	• Respect For Integrity<br>• Reliability<br>• Teamwork<br>• Comfort Promotion
**(57) Saarnio et al. (2017)**	Sweden / Non-profit nursing home	To explore how daily routines and relationships create a sense of "at-homeness" for residents in long-term care.	10 healthcare professionals / Interpretive description	Focus group discussions	Emphasised the importance of continuity, personal knowledge of residents, and relational care practices to maintain dignity and identity.	• Continuity<br>• Personalisation<br>• Relational Care<br>• Resident Identity.

**(58) Bökberg et al. (2019)**	Sweden / Nine nursing homes	To evaluate the effects of staff education on perceptions of person-centred palliative care.	365 healthcare staff / Quasi-experimental design	Structured questionnaires (pre and post-test)	At the initial stage of the study, both groups demonstrated high levels of performance. However, after the intervention, there was only a mild improvement that had been measured. Management and organisational support have been identified as the main factors in sustaining PCC.	• Leadership Engagement<br>• Organisational Support<br>• Team Culture.
**(59) Schenell et al. (2020)**	Sweden / Forty residential care facilities	To co-develop a model that promotes self-determination and autonomy in residential care.	Mixed group (staff, residents, managers) / Participatory design	Focus groups and workshops	The "In my way, at my pace, with the help of you" model was produced, with a view to reinforcing relational autonomy through trust, respect and shared decision-making.	• Autonomy<br>• Trust<br>• Empowerment<br>• Collaborative Care.
**(60) Kastbom et al. (2019)**	Sweden / Nine nursing homes	To explore healthcare professionals' experiences with advance care planning (ACP).	25 professionals (physicians, nurses) / Qualitative study	Individual interviews	Four phases of ACP identified: exploring preferences, integrating views, documenting, and revisiting plans. Highlighted the need for collaborative ACP and clear communication.	• Advance Care Planning; Communication<br>• Ethical Discussion.
**(61) Doody et al. (2024)**	Ireland / Twenty-five nursing homes	To examine perceptions of residents, relatives, and staff on palliative and person-centred care provision.	118 participants (residents, family members, staff) / Quantitative cross-sectional study	Structured questionnaires	Reported overall positive experiences but noted communication gaps and resource limitations. Underlined the ethical duty to uphold residents' and professionals' human rights.	• Communication<br>• Human Rights<br>• Ethical Leadership<br>• Staff Education.
**(54) Mikaelsson et al. (2025)**	Sweden / Five residential care homes	To explore residents' and families' experiences of proactive end-of-life conversations.	11 residents, 8 relatives / Qualitative interview study	Semi-structured interviews	Identified benefits such as open dialogue, shared understanding, emotional security, and relationship strengthening. Promoted early discussions as a core element of PCC.	• Open Communication<br>• Trust<br>• Family Involvement<br>• Emotional Preparedness.

Table 2 – Analysis of the selected paper
