# Supplementary material for: Person-centred care models for institutionalised older adults requiring palliative care: an integrative review
Source: BMC Geriatr. 2026 Jul 7;26:1235. doi: 10.1186/s12877-026-07795-y (PMC13628849; doi:10.1186/s12877-026-07795-y)
Supplement: Supplementary file 1 [file 12877_2026_7795_MOESM1_ESM.zip › Material suplementar /Supplementary File 2 ΓÇô PRISMA 2020 Checklist .pdf]

PRISMA 2020 Checklist

Section and Topic	Item #	Checklist item	Location where item is reported
**TITLE**			
Title	1	Identify report as a systematic/integrative review	P1 Integrative Review
**ABSTRACT**			
Structured summary	2	Provide a structured summary including background; objectives; data sources; study eligibility criteria, participants; study appraisal and synthesis methods; results; limitations; conclusions and implications of key findings; systematic review registration number.	P2
**INTRODUCTION**			
Rationale	3	Describe the rationale for the review in the context of existing knowledge.	P3-5
Objectives	4	Provide an explicit statement of the objective(s) or question(s) the review addresses.	P3-5
**METHODS**			
Protocol and registration	5	If review protocols exist, the relevant details should be indicated, including where they can be accessed (e.g. web address). If available, registration information should be provided, including the registration number.	P6
Eligibility criteria	6	Specify study characteristics (e.g. years considered, language, publication status) used as eligibility criteria, giving rationale.	P6-7
Information sources	7	Specify all databases, registers, websites, organisations, reference lists and other sources searched or consulted to identify studies. Specify the date when each source was last searched or consulted. (See Supplementary Materials)	P6
Search strategy	8	Present the full search strategies for all databases, registers and websites, including any filters and limits used. (See Supplementary Materials)	P7<br>Supplementary File 3<br>(Search Strategy and Data Extraction Table)
Selection process	9	Specify the methods used to decide whether a study met the inclusion criteria of the review, including how many reviewers screened each record and each report retrieved, whether they worked independently, and if applicable, details of automation tools used in the process. (See Supplementary Materials)	P8-9; Figure 1<br>(PRISMA Flow Diagram)
Data collection process	10	Specify the methods used to collect data from reports, including how many reviewers collected data from each report, whether they worked independently, any processes for obtaining or confirming data from study investigators, and if applicable, details of automation tools used in the process.	P9
Data items	11	List and define all outcomes for which data were sought. Specify whether all results that were compatible with each outcome domain in each study were sought (e.g. for all measures, time points, analyses), and if not, the methods used to decide which results to collect. (See Supplementary Materials)	P9
Study risk of bias assessment	12	The methodological quality and risk of bias of individual studies were assessed using the Joanna Briggs Institute (JBI) Critical Appraisal Checklist for qualitative and quantitative research.	P9 Supplementary File 1 (JBI Critical Appraisal table)
Summary Measures	13	This review employed a narrative and thematic synthesis due to methodological heterogeneity across included studies. (See Supplementary Materials)	P10–11; Supplementary File 3 (Narrative and Thematic Synthesis Summary)

PRISMA 2020 Checklist

Section and Topic	Item #	Checklist item	Location where item is reported
Synthesis of Results	14	Data were synthesised narratively through thematic grouping of study findings. Quantitative synthesis (meta-analysis) was not performed due to methodological heterogeneity	P10-13<br>Table 2 (analysis of the Selected Papers)
Risk of Bias Across Studies	15	Risk of bias was assessed qualitatively using the JBI checklist; no statistical analysis of bias was performed due to the narrative synthesis.	P9<br>File 1
Additional Analyses	16	Statistical analyses; thematic comparisons used to explore consistency across studies. (See Supplementary Materials)	P8; Figure 1 and Supplementary File 4
**RESULTS**			
Study selection	17	The search and selection process is described, including details of the number of records identified, duplicates removed, and studies ultimately included. This process is illustrated in the PRISMA 2020 flow diagram.	P8–10; Table 2 and Supplementary File 3
Study characteristics	18	The main characteristics of each included study are synthesised, including information on the country, setting, aims, participants, design and data collection method. (See Supplementary Materials)	P10-13<br>Table 2 (analysis of the Selected Papers)
Risk of bias in studies	19	Methodological quality and potential bias were assessed for each study using the Joanna Briggs Institute (JBI) Critical Appraisal Checklist. Findings from this assessment informed the synthesis and interpretation of results.	P9
Results of individual studies	20	The results and conclusions of the individual studies are presented in a narrative form and summarised. Quantitative estimates or meta-analyses were not applicable	P12-13
Results of syntheses	21	The synthesis methods and summary presentation of findings are described; no quantitative pooling or subgroup analyses were performed. (See Supplementary Materials)	P13-17<br>Discussion (Summery of Evidence)
Reporting biases	22	Reporting bias was considered through methodological triangulation and transparent inclusion criteria. No evidence of selective reporting was detected.	P9
Certainty of evidence	23	The certainty of evidence was discussed narratively, considering methodological quality, sample variation, and study consistency.	P14-17<br>Discussion (Summery of Evidence)
**DISCUSSION**			
Summary of Evidence	24	The discussion summarises the main findings and situates them within the broader literature on person-centred and palliative care for institutionalised older adults. The overall evidence is discussed in relation to its relevance for practice, education and policy.	P14-17
Limitations	25	The review acknowledges several limitations, including the small number of studies, methodological heterogeneity, predominance of research from Sweden, and limited exploration of residents' and families' perspectives.	P18
Conclusions	26	The review concludes that person-centred and palliative care principles are essential to improving care quality and guiding future research and policy.	P19

PRISMA 2020 Checklist

Section and Topic	Item #	Checklist item	Location where item is reported
**OTHER INFORMATION**			
Funding	27	No specific funding was received. The funders had no role in study design, data collection, analysis, or interpretation. (See Supplementary Materials)	P21<br>Supplementary Materials section

From: Page MJ, McKenzie JE, Bossuyt PM, Boutron I, Hoffmann TC, Mulrow CD, et al. The PRISMA 2020 statement: an updated guideline for reporting systematic reviews. BMJ 2021;372:n71. doi: 10.1136/bmj.n71. This work is licensed under CC BY 4.0. To view a copy of this license, visit https://creativecommons.org/licenses/by/4.0/
